# High germline mutation rates, but not extreme population outbreaks, influence genetic diversity in a keystone coral predator

**DOI:** 10.1371/journal.pgen.1011129

**Published:** 2024-02-12

**Authors:** Iva Popovic, Lucie A. Bergeron, Yves-Marie Bozec, Ann-Marie Waldvogel, Samantha M. Howitt, Katarina Damjanovic, Frances Patel, Maria G. Cabrera, Gert Wörheide, Sven Uthicke, Cynthia Riginos

**Affiliations:** 1 School of the Environment, The University of Queensland, St Lucia, Queensland, Australia; 2 Villum Centre for Biodiversity Genomics, Section for Ecology and Evolution, Department of Biology, University of Copenhagen, Copenhagen, Denmark; 3 Institute of Zoology, University of Cologne, Cologne, Germany; 4 Australian Institute of Marine Science, Townsville, Australia; 5 Department of Earth and Environmental Sciences, Paleontology and Geobiology, Ludwig-Maximilians-Universität München, Munich, Germany; 6 GeoBio-Center, Ludwig-Maximilians-Universität München, Munich, Germany; 7 Staatliche Naturwissenschaftliche Sammlungen Bayerns (SNSB)–Bayerische Staatssammlung für Paläontologie und Geologie, Munich, Germany; University of Rhode Island CELS: University of Rhode Island College of the Environment and Life Sciences, UNITED STATES

## Abstract

Lewontin’s paradox, the observation that levels of genetic diversity (π) do not scale linearly with census population size (*N*_*c*_) variation, is an evolutionary conundrum. The most extreme mismatches between π and *N*_*c*_ are found for highly abundant marine invertebrates. Yet, the influences of new mutations on π relative to extrinsic processes such as *N*_*c*_ fluctuations are unknown. Here, we provide the first germline mutation rate (*μ*) estimate for a marine invertebrate in corallivorous crown-of-thorns sea stars (*Acanthaster* cf. *solaris)*. We use high-coverage whole-genome sequencing of 14 parent-offspring trios alongside empirical estimates of *N*_*c*_ in Australia’s Great Barrier Reef to jointly examine the determinants of π in populations undergoing extreme *N*_*c*_ fluctuations. The *A*. cf. *solaris* mean *μ* was 9.13 x 10^−09^ mutations per-site per-generation (95% CI: 6.51 x 10^−09^ to 1.18 x 10^−08^), exceeding estimates for other invertebrates and showing greater concordance with vertebrate mutation rates. Lower-than-expected *N*_*e*_ (~70,000–180,000) and low *N*_*e*_/*N*_*c*_ values (0.0047–0.048) indicated weak influences of population outbreaks on long-term π. Our findings are consistent with elevated *μ* evolving in response to reduced *N*_*e*_ and generation time length, with important implications for explaining high mutational loads and the determinants of genetic diversity in marine invertebrate taxa.

## Author summary

Understanding how levels of genetic diversity levels are maintained in natural populations and their relationship to species abundance is central to many conservation problems. We address this question in ecologically important crown-of-thorns sea stars to advance our understanding about the determinants of genetic diversity in species undergoing extreme population size fluctuations or *outbreaks*. In this work, we report the first germline mutation rate estimate for a marine invertebrate using whole-genome sequencing of parent-offspring trios. Our results reveal that unexpectedly high mutational contributions and reduced effective population size (stronger genetic drift than predicted by abundance) shape genetic diversity in this species, despite outbreaking populations sizes exceeding 20–90 million individuals. Our results are consistent with theory on mutation rate evolution, whereby elevated mutation rates evolve in response to reduced effective population size or generation time length. Our findings highlight the potential importance of high mutation rates in maintaining extreme deleterious mutational loads observed in marine invertebrate taxa and moderate genetic diversity levels despite population declines. Such fundamental knowledge advances our understanding about the determinants of genetic diversity in large marine populations and is valuable for testing future hypotheses on mutation rate evolution across diverse animal phyla.

## Introduction

Understanding how and why genetic diversity varies among taxa is a longstanding evolutionary puzzle [1]. The neutral theory of evolution predicts that genetic diversity should increase proportionally with effective population size, *N*_*e*_ [2], where the expected level of pairwise genetic diversity (π) at neutral sites reflects the balance between new mutations and loss via genetic drift (as reflected by *N*_*e*_) and can be approximated by π *= 4N*_*e*_*μ*, where *μ* is the mutation rate per-base pair, per-generation. However, empirical evidence shows that the range of π observed in natural populations does not scale with ranges in census population sizes (*N*_*c*_) [1,3,4]. This disparity between π and *N*_*c*_ variance across taxa, known as Lewontin’s paradox, contradicts theoretical expectations and challenges our understanding of how natural variation is maintained. With increasing evidence that genetic diversity is central to many conservation problems [5], including inferences about extinction risks and species responses to environmental change, our ability to understand how diversity levels are maintained and how they relate to census population sizes is critical. Yet, the determinants of π and the causative factors underlying Lewontin’s paradox are still debated well into the genomic era [4,6–9].

**Fig 1:**
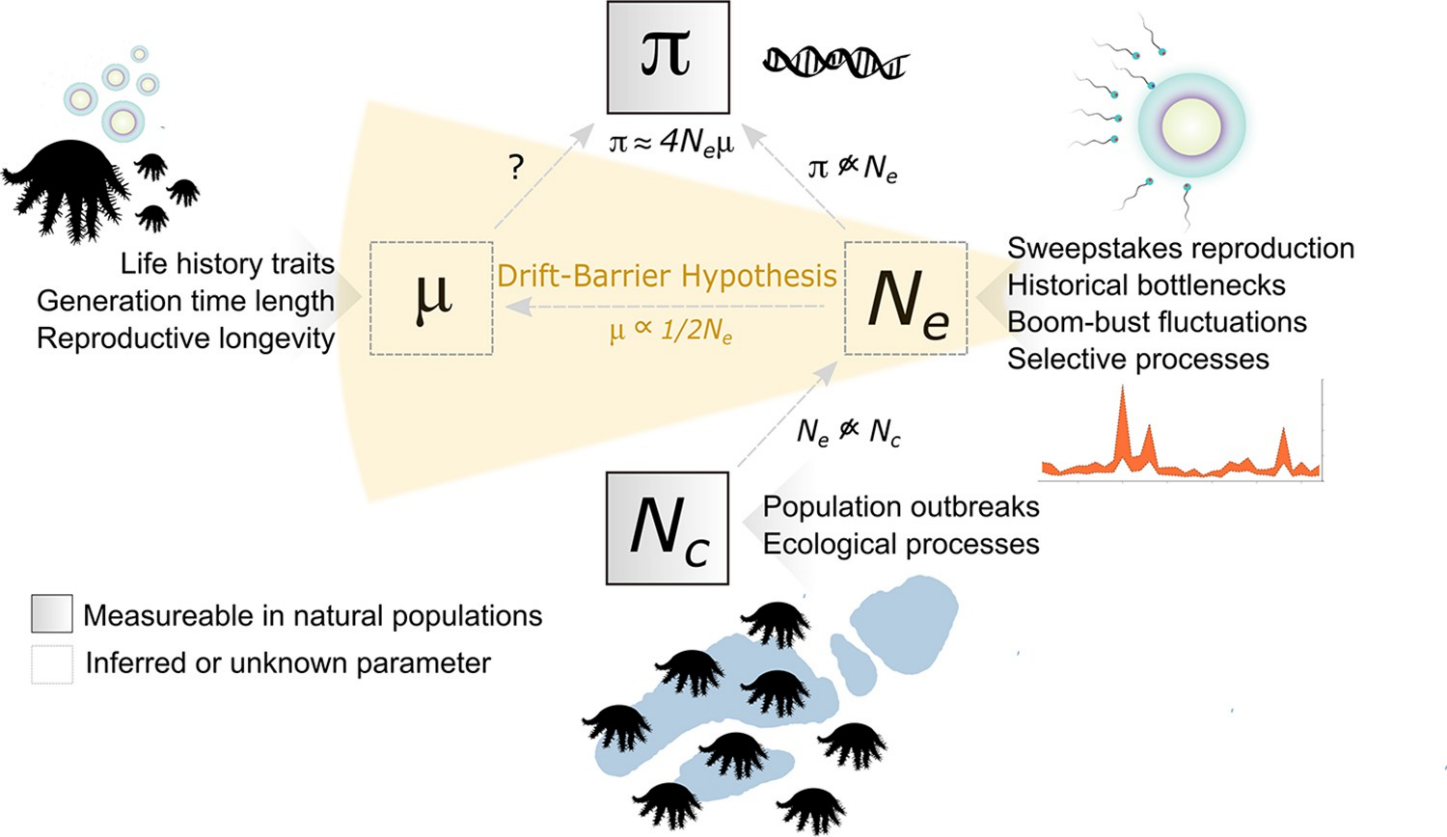
Conceptual diagram illustrating the relationships between evolutionary parameters underlying genetic diversity. Under mutation-drift equilibrium, pairwise genetic diversity (π) reflects the balance between new mutations (*μ*) and the loss of variation via genetic drift, reflected by effective population size (*N*_*e*_). In natural populations, π does not increase linearly with *N*_*e*_ and *N*_*e*_ is often smaller than census population size (*N*_*c*_). *μ* is unknown for marine invertebrate taxa, thus the contributions of new mutations to π remain unknown. The drift-barrier hypothesis, a leading explanation for *μ* variation, proposes that selection against high *μ* is less efficient in small *N*_*e*_ species. This leads to an inverse relationship between *μ* and *N*_*e*_ (*μ* ~1/2*N*_*e*_ for diploid organisms) and the evolution of high *μ* in small *N*_*e*_ populations. Key evolutionary and ecological processes or traits affecting the magnitude of each parameter are shown. Several evolutionary processes act in combination to reduce *N*_*e*_ and thus, constrain π, decoupling it from *N*_*c*_. This decoupling leads to a disparity between the range of π and *N*_*c*_ variance observed across taxa, known as Lewontin’s paradox. π can be measured in natural populations using DNA sequencing to calculate pairwise differences between sampled individuals and *N*_*c*_ can be approximated most accurately from direct observations of organisms from field surveys. *μ and N*_*e*_ are inferred parameters from polymorphism data.

Population genetic studies aiming to solve Lewontin’s paradox suggest that neutral and selective processes act in combination to decouple π from *N*_*c*_ [9] (Fig 1). Historical demographic fluctuations and contemporary dynamics such as repeated extinctions and recolonisations or pest population outbreaks may result in founder events that substantially reduce *N*_*e*_ [10–12]. Positive selection [13] and the indirect effects of background selection and recombination rate variation may also reduce linked neutral diversity to levels lower than predicted by *N*_*c*_ [9,14,15], where the effect of these selective processes on π is expected to be greater in large populations [6,8]. Importantly, equilibrium values of π are also a function of the mutation rate, *μ*, which varies by orders of magnitude across animal taxa [16]. Several hypotheses seek to explain *μ* variation across organisms. In particular, the drift-barrier hypothesis proposes that more efficient selection against high mutation rates (relative to genetic drift) in large populations leads to maximal fine-tuning of DNA repair and replication mechanisms and predicts that reduced germline mutation rates should evolve in highly abundant taxa [17–19] (Fig 1). Alternatively, life history traits such as differences in generation time length [16] and reproductive longevity [20,21] may be important determinants of *μ* evolution across animal phyla, whereby longer-lived organisms have higher mutation rates compared to shorter-lived taxa (which also tend to be more abundant). While these predictions help explain lower-than-expected π in highly abundant species, the extent to which *μ* variation influences π relative to extrinsic processes remains unknown for most taxa.

Bentho-pelagic marine organisms have long been recognised for harbouring extreme levels of genetic diversity (π ~2–8% [3,4,22,23]). For example, prolific fecundity and long-lived planktonic larval stages, coupled with few physical barriers to gene flow in the marine environment can enable the establishment of large and genetically diverse populations [24]. While the assumption of large population sizes holds true for some marine taxa as a possible explanation for high π [25], marine invertebrates show some of the most extreme mismatches between π and *N*_*c*_ towards both lower [26] and higher relative genetic diversity [4,8]. High variance in larval recruitment and reproductive success (i.e., sweepstakes reproduction) among broadcast spawning adults may drastically decrease *N*_*e*_ relative to population size [27]. Similarly, the ‘boom-bust’ population ecologies of many marine invertebrates, whereby populations rapidly increase in density followed by dramatic declines [28,29], can decouple *N*_*e*_ from *N*_*c*_ (Fig 1). Theory suggests that long-term *N*_*e*_ in fluctuating populations is approximated by the harmonic mean and thus bound closer to population sizes during contraction periods [10,30,31]. From the other perspective, exceptionally high π in highly fecund and dispersive species (e.g., tunicates and mussels, [4,25,32,33]) exceeds expectations based on approximated *N*_*c*_ (see Fig 2 in [8]). In both cases, decomposing the determinants of π (*N*_*e*_ and *μ*) and their relationship to *N*_*c*_ provide deeper insights regarding the processes maintaining genetic variation in natural populations [34].

**Fig 2:**
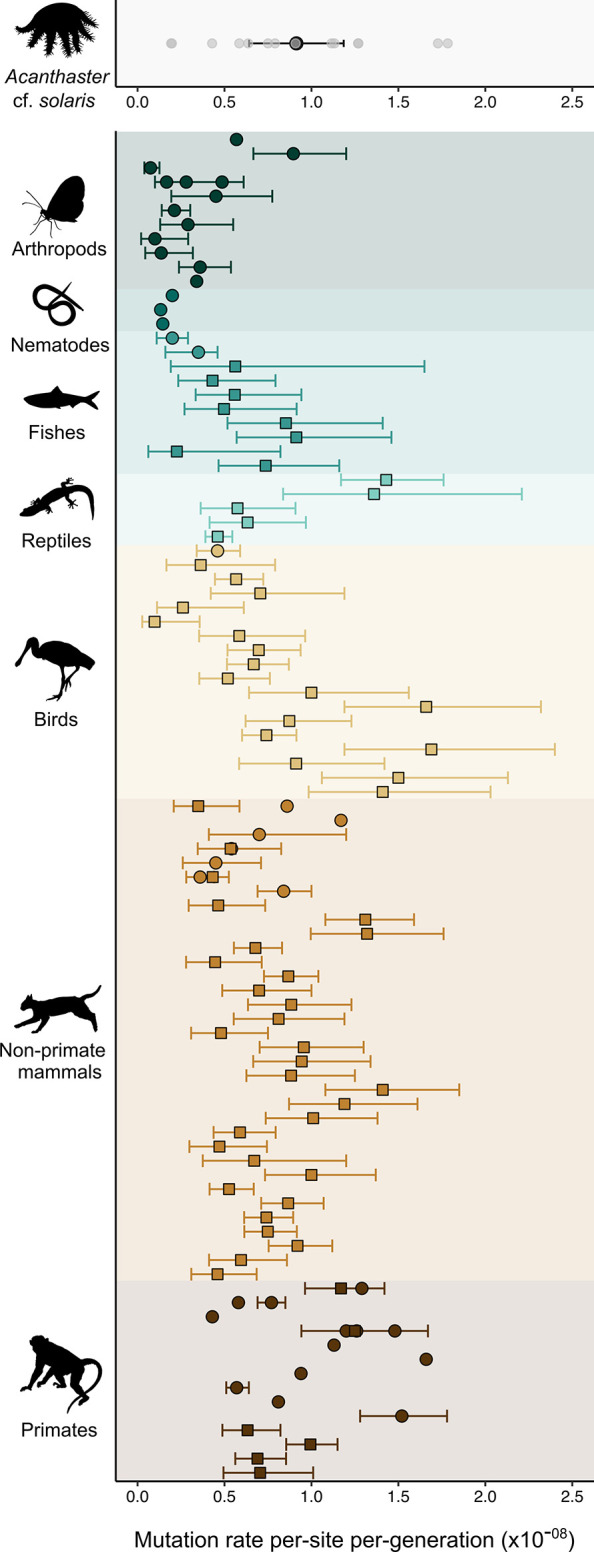
Germline mutation rates inferred in *Acanthaster* cf. *solaris* and other metazoan taxa. Top panel: Germline mutation rates (*μ*) for 14 *A*. cf. *solaris* parent-offspring trios. Individual estimates for each trio are shown in grey. The average *μ* is show in black and 95% confidence intervals are indicated with error bars. Lower panel: Previously published average *μ* estimates and 95% confidence intervals for representative metazoan groups. Mutation rates estimates from [16] are represented by squares and estimates from other publications (both pedigree-based and mutation accumulation approaches) are represented by circles. 95% confidence intervals are shown as reported or omitted if not reported. In cases where multiple estimates are available for a single taxon, 95% confidence intervals from the most recent publication are shown. Refer to S9 Table for article details. Exceedingly high mutation rate estimates (*μ* > 2.5 x 10^−08^) for *Thamnophis sirtalis* and *Rhea pennata* [16] (S9 Table) are omitted for visual purposes only. Silhouettes were sourced from Phylopic (http://phylopic.org).

Focusing on how *μ* affects π, determining whether high contributions of new heritable mutations underlie high π values could help explain how extreme polymorphism levels are maintained in some marine invertebrate taxa (e.g., genome-wide silent site π ~ 8.0% for *Bostrycapulus aculeatus*, [4] and *Ciona savignyi*, [3]; π ~ 5.0% for *Ciona intestinalis*, [33]; π ~ 2.3% for *Crassostrea gigas*, [23]). Marine invertebrates also show some of the highest reported rates of deleterious recessive mutations associated with early mortality (i.e., high mutational load) for animal taxa, exemplified by wild populations of mussels, oysters and abalones (e.g., [35–38]). Thus, one possible explanation for large mutational loads in marine invertebrates is that their genome-wide mutation rates are substantially higher than terrestrial animals and marine fishes [39]. Indirect measures of mutational load based on comparisons of synonymous and nonsynonymous diversity also support elevated genomic mutation rates in the marine invertebrate taxa studied (e.g., [33,37]). Despite the importance of quantifying new mutational contributions to genetic diversity, little empirical data exists on germline spontaneous mutation rates for marine organisms. The current knowledge for germline mutation rates in marine metazoans is limited to five fish taxa [16,40], and there are no direct estimates of germline mutation rates for marine invertebrates.

In this study, we decompose key evolutionary parameters (*μ*, *N*_*e*_, *N*_*c*_) in one of the world’s most intensely monitored marine invertebrate species, the corallivorous crown-of-thorns sea star (CoTS; *Acanthaster* cf. *solaris*) to advance our understanding about the determinants of π in populations undergoing extreme *N*_*c*_ fluctuations. (Fig 1). We present the germline mutation rate, *μ*, for *A*. cf. *solaris* using deep sequencing of pedigree trios of wild-caught parents and early-stage larval offspring, alongside detailed estimates of *N*_*c*_ calculated from 32 years of long-term field monitoring and high-resolution mapping of reef geomorphology in Australia’s Great Barrier Reef (GBR). CoTs in the genus *Acanthaster* are diploid, broadcast spawning asteroid echinoderms that reach sexual maturity at about two years of age when they become corallivorous [41,42]. Although CoTS are native to the Indo-Pacific Ocean, they are among the most influential keystone predators in tropical coral reef ecosystems. CoTS populations can locally reach massive adult densities during cyclical outbreak events (>1,500 individual ha^-1^) [43,44] during which populations can decimate coral reefs [45]. In the GBR, predation by outbreaking *A*. cf. *solaris* populations is among the leading causes of hard coral cover loss [45,46]. Population outbreaks are followed by rapid declines to population densities several orders of magnitude below outbreaking densities within a few years (~3 individual ha^-1^) [47,48]. Such boom-bust population fluctuations appear characteristic for echinoderm taxa and are also common to many pests and invasive species. However, empirical genomic datasets for fluctuating or outbreaking populations are rare, limiting our knowledge about the impact of population fluctuations on π and as a contributing factor to extreme cases of Lewontin’s paradox [9].

Our first aim is to directly quantify *μ* and infer *N*_*e*_. Given the inverse predicted relationship between *μ* and *N*_*e*_ (i.e., drift-barrier hypothesis), we expect that *A*. cf. *solaris μ* is low and similar to values reported for other highly abundant invertebrates, where presumably large *N*_*e*_ favours the evolution of low mutation rates. Alternatively, if *N*_*e*_ is low, or if elevated *μ* underlies high mutational loads [39] and extreme polymorphism levels observed in some marine invertebrates (e.g., [4]), we may expect that *A*. cf. *solaris μ* is high relative to terrestrial animals and marine fishes. Similarly, if generation time length is an important determinant of mutation rate variation, the *A*. cf. *solaris μ* may exceed values reported for annual, shorter-lived invertebrate taxa, such as insects, and align closely with *μ* estimates for longer-lived organisms. *A*. cf. *solaris* are one of the most intensely monitored wild marine invertebrate taxa in the world providing far greater resolution on *N*_*c*_ than is typically feasible for marine invertebrate taxa, where indirect approaches for estimating *N*_*c*_ have been based on body size predictors of population density and distribution ranges extracted from publicly-sourced occurrence data (e.g., [6,8]). Thus, our second aim is to clarify the relationship between *N*_*e*_ and *N*_*c*_ with higher certainty to provide insight into the magnitude of genetic drift influencing π in large populations experiencing frequent outbreaks. Our study provides the first germline mutation rate estimate for a marine invertebrate, a crucial basis for interpreting genetic diversity patterns within species [17], and advances our understanding of the processes controlling levels of natural genetic variation in an ecologically significant taxon.

## Methods and materials

### Parental gonad collection and fertilisation

Reproductively mature *A*. cf. *solaris* were collected from John Brewer Reef (18°38’S, 147°03’E) in the Great Barrier Reef (GBR; November 2020). Specimens used for the analysis were collected by the Great Barrier Reef Marine Park Authority’s (GBRMPA) CoTS control program and were thus from areas with densities above the culling threshold (e.g., 10–15 individuals ha^−1^; [49,50]). Collection of gonad tissue, in vitro breeding experiments and larval rearing were performed at the National Sea Simulator (SeaSim) Experiment Rooms at the Australian Institute of Marine Science (AIMS; Townsville, Queensland) following procedures outlined in [51]. However, here we fertilised individual males and females as unique parental pairs and raised their embryos and larvae for 3–8 days. For each parental cross, we selected two larvae in the early-mid bipinnaria stage (day 3–8) or early-mid brachiolaria stage (day 8–9) (Fig A in S1 Text). DNA from individual larvae was extracted using the QIAGEN Blood and Tissue kit and following modifications for *A*. cf. *solaris* larvae from [52]. Adult DNA was extracted from gonad tissue using a modified CTAB protocol optimised for marine invertebrate tissue [53] and purified with PCR-DX Clean beads. Whole genome libraries were prepared for seven biparental families with two offspring per family to generate 14 mother-father-offspring trios including siblings. Genomic libraries were sequenced on a single lane of the NovaSeq S4 300 cycle to achieve 60X coverage using 2 x 150 bp paired reads. Refer to S1 Text for additional details on breeding experiments, DNA extraction and genomic library preparation.

### Genomic data processing, variant calling and site selection

Raw reads were quality filtered using Trimmomatic (v0.39) [54]. Quality-filtered reads were mapped to the *A*. cf. *solaris* reference genome (GCF_001949145.1_OKI-Apl_1.0) [55] using the Burrow-Wheeler Aligner (v0.7.17 BWA) [56]. BAM files were sorted and indexed using Samtools v1.10 [57] and PCR duplicates were removed using picard (http://broadinstitute.github.io/picard/). The bioinformatic pipeline used for calling germline mutations from pedigree samples was initially described in [58] and follows best practices principles outlined in [59]. All scripts are available on Github (https://github.com/lucieabergeron/germline_mutation_rate). Variant calling was performed in GATK (v4.0.7.0) using HaplotypeCaller (BP-RESOLUTION) constrained to 693 scaffolds greater than 10,000 bp. Individual gVCF files were collated into a GenomicsDB for each trio using GenomicsDBImport and joint genotyping was performed using GenotypeGVCF. SNPs were filtered to remove sites following site-specific parameters: QD < 2.0; FS > 20.0; MQ < 40.0; MQRankSum < -2.0, MQRankSum > 4.0, ReadPosRankSum < -3.0, ReadPosRankSum > 3.0, SOR > 3.0. Refer to S1 Text for additional details on genomic data and variant filtering.

### Detecting and filtering of *de novo* mutations

We detected candidate *de novo* mutations (DNMs) as positions with Mendelian violations using GATK SelectVariants, where one of the alleles observed in an offspring is not present in either parent. We retained sites in which: (i) parents were homozygous for the reference allele and the offspring was heterozygous (parental alternative allelic depth per site, AD = 0); (ii) all individuals within a trio have GQ > 70, DP > 0.5 * average individual depth, and DP < 2 * average individual depth; and (iii) the offspring had an allelic balance (AB) between 0.3 and 0.7. We recalled regions with candidate DNMs with bcftools (version 1.2) [60] and classified any candidates not jointly detected by GATK and bcftools as False Positive (FP) DNMs. All resulting DNMs and mutation-associated regions were manually inspected using original BAM files in Integrative Genomics Viewer [61]. We ruled out mis-mapping errors, validated homozygous parental genotypes and confirmed shared DNMs between siblings following six criteria to assign DNMs as spurious (S1 Text). Refer to S1 Text for details on DNM detection and manual curation.

### Germline mutation rate estimation

To estimate the germline mutation rate, we estimated the genome portion for which we had power to detect candidate DNMs, considering all sites where mutations could be detected (i.e., number of callable sites) and corrections for the false negative rate (S1 Text). This was done by selecting every position in the VCF files (BP_RESOLUTION output) for which both parents were homozygous for the reference allele and all three individuals passed GQ and DP filters (as described above) [58]. The mutation rate was then estimated for each trio as:

μ=(nb_DNM–nb_FP)/(2*C*(1‐FNR)),

where nb_DNM is the number of DNMs, nb_FP is the number of false positive mutations, C is the number of callable sites in the genome and FNR is the false negative rate.

### Parental origins and mutation characteristics

We phased DNMs to their parental origins using a read-back phasing approach [62] (https://github.com/besenbacher/POOHA) to determine the proportion of male-to-female contributions (α) to DNMs. DNMs were classified by mutation type and mutations resulting in a change from C to any base were classified as CpG sites. We annotated variants and predicted their genomic location with snpEff v5.1 [63] according to the *A*. cf. *solaris* reference genome annotations [55] to assess whether the number of DNMs in each annotation category was significantly greater than expected by chance (S1 Text).

### Effective population size (N_e_) estimation

We used our new *μ* estimate and population nucleotide diversity (π) across 14 parental genomes using ANGSD (v0.934) [64] to calculate effective population size as *N*_*e*_ *=* π */(4μ)*. It is important to note that population genomic programs leveraging whole genome data (e.g., ANGSD [64] and Sequential Markovian coalescent approaches [65]) often rely on SNPs for inference because their mutational process is well approximated by the infinite sites model [66]. Neglecting sites with multiple mutations (e.g., tri-allelic SNPs) can bias diversity estimates [67] and may be especially relevant for high diversity species (π >>0.05) in which the infinite sites model is violated and π is no longer proportional to *N*_*e*_ [68]. Because our dataset of filtered variants (quality thresholds as applied to GONE analyses described below) across 14 parental genomes did not contain sites with multiple alleles, it is reasonable to assume that no or very few sites show multiple mutations affecting diversity and *N*_*e*_ estimates in *A*. cf. *solaris*.

We inferred historical changes in *N*_*e*_ (> 10,000 years ago) using Multiple Sequential Markovian Coalescent (MSMC2) analysis [65,69] The MSMC method approximates the most recent time to coalescence between haplotypes across multiple diploid phased genomes, where the coalescence rate distribution is used to infer changes in historical *N*_*e*_ over an extended time period into the past (several hundreds to thousands of generations) [65,70]. For this analysis, we adapted scripts from Github (https://github.com/iracooke/atenuis_wgs_pub) [71]. MSMC2 analyses were executed for each pair of phased parental genomes (4 haplotypes) using a single randomly chosen offspring for phasing. A distribution of *N*_*e*_ was obtained for each parental pair applying our inferred mean *μ* and a 2 year generation time as inferred from laboratory and field studies in *A* cf. *solaris* [41,42,72].

To infer *N*_*e*_ on recent timescales (< 100 generations ago) and to generate *N*_*e*_ estimates that are independent of our inferred *μ*, we used the Genetic Optimisation for *N*_*e*_ Estimation (GONE) method [73]. The GONE method leverages the distribution of linkage disequilibrium (LD) between pairs of loci over a range of recombination rates from SNP data to infer recent *N*_*e*_ changes (0–200 generation). Because LD patterns between pairs of loci at various genetic distances provide *N*_*e*_ information at different time points in the recent past, the GONE genetic algorithm can infer the recent *N*_*e*_ trajectory that best explains observed LD patterns, providing much greater resolution on contemporary *N*_*e*_ not captured by coalescent-based methods [70,73]. For this analysis, we used the 14 parental genomes, retained scaffolds greater than 1M bp and variants passing minimum quality criteria (S1 Text). We used the default recombination rate of 1 centimorgans per megabase and a maximum recombination rate of 0.01 (hc = 0.01) as recommended by the authors [73]. Because the GONE method considers the compounded effects of genetic drift from all previous generations, we calculated the arithmetic mean *N*_*e*_ between the last 10–80 generations to exclude the most recent and distant generations where estimation may not be reliable [73]. Refer to S1 Text for details on *N*_*e*_ estimation.

### Census population size (*N*_*c*_) estimation using long-term monitoring data

We inferred the size of the contemporary *A*. cf. *solaris* population in the GBR Marine Park using three decades of benthic monitoring data [74] combined with high-resolution (10 m) mapping of the reef geomorphology of the GBR to estimate the area of suitable *A*. cf. *solaris* habitat [75,76]. This mapping product characterises the geomorphic zonation of 2,164 offshore reefs [75] and 890 fringing and nearshore reefs [76] of the GBR Marine Park to a 20 m depth using classifications of physical attributes derived from remote-sensing data (sub-surface reflectance, bathymetry, slope angle) and wave modelling. As representative *A*. cf. *solaris* habitats, we only considered geomorphic categories predominantly covered by consolidated hard substrate, which is more suitable for coral colonisation. According to Roelfsema et al. [75], there are 4 geomorphic categories that are representative of significant ‘coral habitat’: ‘outer reef flat’, ‘reef slope’, ‘reef crest’, and ‘shelter reef slope’. These habitats are likely to support the greatest share of adult *A*. cf. *solaris* populations, as they provide optimal conditions for abundant shelter and food source. Refer to S1 Text for additional details. The available census data were collected using standardised benthic surveys performed between 1991 and 2022 on a selection of individual reefs. For each surveyed reef, individual counts were converted into an estimate of non-cryptic *A*. cf. *solaris* density (i.e., adult density, where adults are approximately > 15 cm diameter) (Fig B in S1 Text), and a mean value of reef-level density (individuals km^-2^) was calculated for each annual sample of monitored reefs. A 95% confidence interval of annual mean densities was calculated from 500 pseudo-samples generated by bootstrap for each monitored year. Finally, the confidence limits and the mean of the annual mean densities were multiplied by the total surface (3D) area of the preferred *A*. cf. *solaris* habitat of reef-building corals on the GBR (14,199 km^2^, S1 Text).

## Results

### Germline mutation rate in *A*. cf. *solaris*

High-coverage sequencing of 14 *A*. cf. *solaris* parent-offspring trios resulted in an average coverage of 59X per individual after mapping (range 37X to 91X) (S1 Table). Analyses of relatedness validated trio parent-offspring relationships (relatedness_phi ~0.25 between parents and offspring) and low parental relatedness (relatedness_phi < 0.015 between parents) (S2 Table). Variant calling in GATK resulted in 7,316,821 SNPs post-filtering (mean across trios) and 596 DNM candidates based on Mendelian violations (S3 and S5 Tables). Additional filtering for sites with no reads supporting parental alternative alleles (AD = 0) resulted in 246 variants (S4 and S5 Tables). Following variant calling with an independent approach, 141 of 246 variants passed our selection criteria as candidate DNMs. Four false positive mutations were present as low frequency variants in the population genomic dataset spanning the GBR (S6 Table). We further verified 63 out of these 141 mutations (44.7%) as true positive DNMs based on strict manual validation criteria (S6 and S7 Tables). The total number of validated DNMs (n = 63) ranged between 1 and 9 mutations per trio (S5 Table). Approximately 19.0% (12 of 63) of DNMs were shared among siblings where both shared DNMs passed selection criteria.

The average false positive rate was 42% when considering the independent variant calling approach and 31% for manual validation, resulting in a cumulative false positive rate of 73.0% (range 50% to 92% among trios; S5 Table). False positives arose largely from the incorrect absence of a heterozygous site in the parents where 1 or more reads supported the alternative allele. The high rate of false heterozygous genotypes is comparable to other values reported for non-model taxa [16] and could be due to read mapping errors, paralog mis-mapping or incorrect variant calls associated with reference genome quality [77]. The available CoTS reference genome is a scaffold-level assembly, although genome assembly quality is not expected to have large impacts on the accuracy of the mutation rate estimates [16]. Our parent-offspring trios and high coverage genomic data could be used to improve mutation rate estimates in the future with the availability of a chromosome-level assembly for *A*. cf. *solaris* or long-read technologies.

We estimated the per-site per-generation mutation rate as the number of observed true positive DNMs out of the total number of callable sites while accounting for the FNR, estimated to be 8.4% (S5 Table). The number of callable sites ranged from 196,000,000 to 294,000,000 for each trio, representing 71% of the *A*. cf. *solaris* genome on average (S5 Table), which is comparable to other studies (e.g., range for 68 taxa 17–93%, mean = 72%; see Fig S1c in [16]; ~80%; [78]; ~88% [58]). The final estimated mean germline mutation rate averaged across 14 trios was *μ* = 9.13 x 10^−09^ DNMs per-site per-generation (95% CI: 6.51 x 10^−09^ to 1.18 x 10^−08^).

### Parental origins and mutation characteristics

We established parental origins for 53/63 DNMs, with 27 maternally and 26 paternally inherited phased DNM across all trios (S7 and S8 Tables). The ratio of male-female contributions (α) to DNMs was 0.96, not statistically distinguishable from 1 (Welch two sample t-test; p = 0.50; Fig CA in S1 Text). There was no effect of family grouping on between group variance (ANOVA; p = 0.33; Fig D in S1 Text). Four phased pairs of DNMs shared among siblings showed no parental bias, however the remaining two pairs of shared DNMs could not be phased to parental origins. Out of 63 DNMs, the number of transitions (n = 47) exceeded transversions (n = 16) as is typically observed in eukaryotes, yielding a transition to transversion ratio (ti/tv) of 2.94 (Fig CB in S1 Text and S7 Table). 15.9% of DNMs (10 of 63) were located in CpG sites, two of which were classified as missense mutations (Fig CB in S1 Text). Twenty-four DNMs occurred within introns (34.9%) and four DNMs within intergenic regions (6.3%), and nine variants (14.2%) fell within coding regions (missense and synonymous variants) (Fig E in S1 Text and S7 Table). There was no significant enrichment of annotation categories based on genome-wide expectations (p>0.05) after corrections for multiple tests.

### *N*_*e*_ and *N*_*c*_ estimates

**Fig 3:**
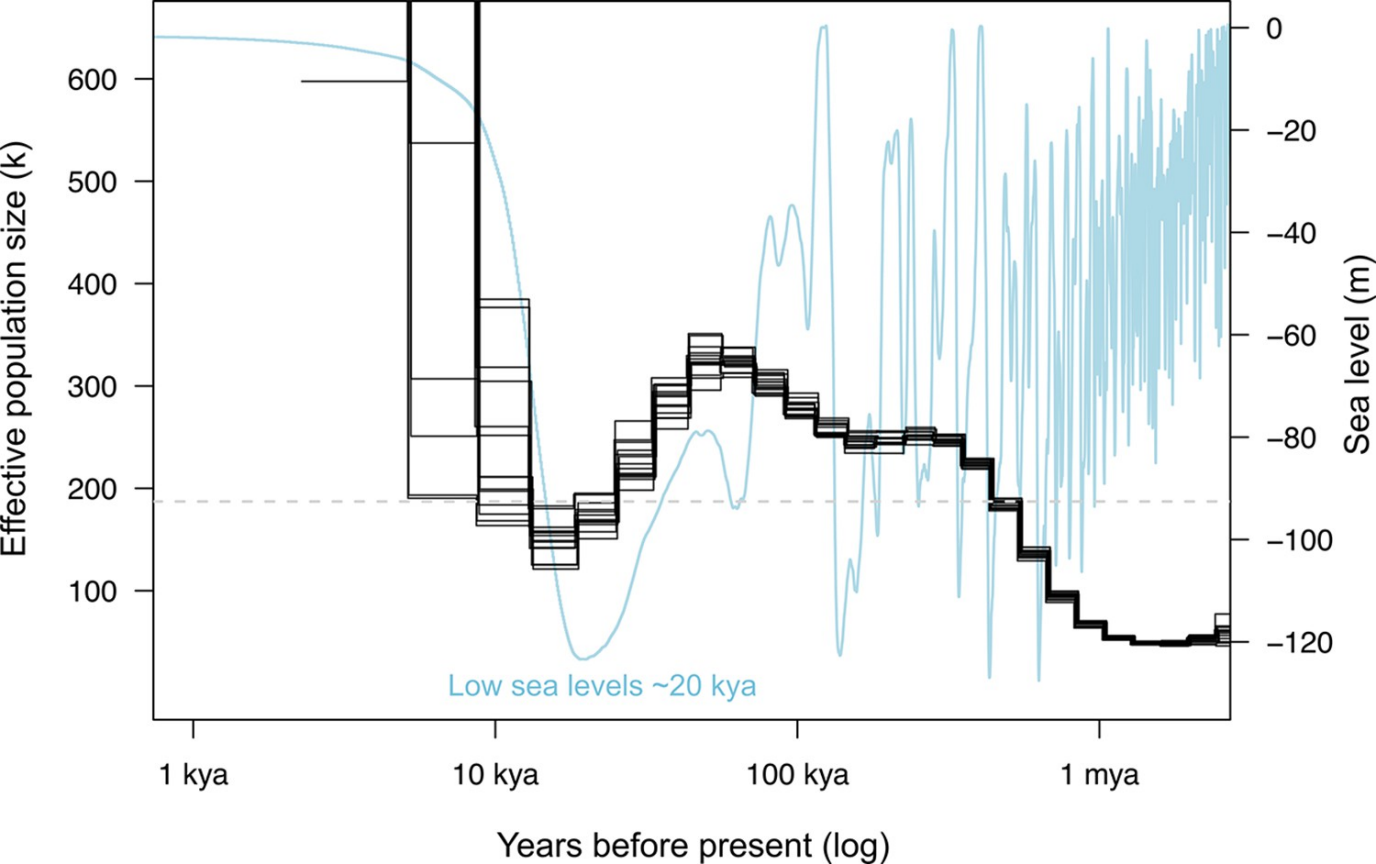
Historical *N*_*e*_ trajectories inferred in MSMC2 applying the inferred *Acanthaster* cf. *solaris* mutation rate and nucleotide diversity from 14 parental genomes (four parental haplotypes per larva). Populations have experienced large fluctuations over the past 10,000 to 1 million years, with peak population sizes ~60,000 years ago and a most recent minimum ~20,000 years ago coinciding with the lowest global sea levels during the late Pleistocene [79]. The long-term harmonic *N*_*e*_ between the last 10,000 to 1 million years (~180,000) is indicated by the grey dashed line.

Based on our estimated *μ* and π from 14 parental individuals (π = 0.0108), we calculated the long-term *N*_*e*_ (π*/4μ*) of *A*. cf. *solaris* to be 296,000 based on equilibrium expectations. Historical *N*_*e*_ trajectories inferred by MSMC2 using four parental haplotypes (per larva) showed peak *N*_*e*_ values ~60,000 years ago, followed by a decline to a most recent minimum ~20,000 years ago, coinciding with the lowest global sea levels during the last glacial maximum (data from [79]; Fig 3). However, recent *N*_*e*_ estimates (< 10,000 years) are likely inflated and less reliable because there are few coalescent events expected to have occurred on these recent timescales [65]. The historical harmonic mean *N*_*e*_ over the last 10,000 to 1 million years was 187,141 and 238,656 over a more recent time period (10,000–100,000 years). Estimates of recent *N*_*e*_ using GONE that are independent of our inferred *μ* returned a mean *N*_*e*_ of 67,755 between the last 10–80 generations.

**Fig 4:**
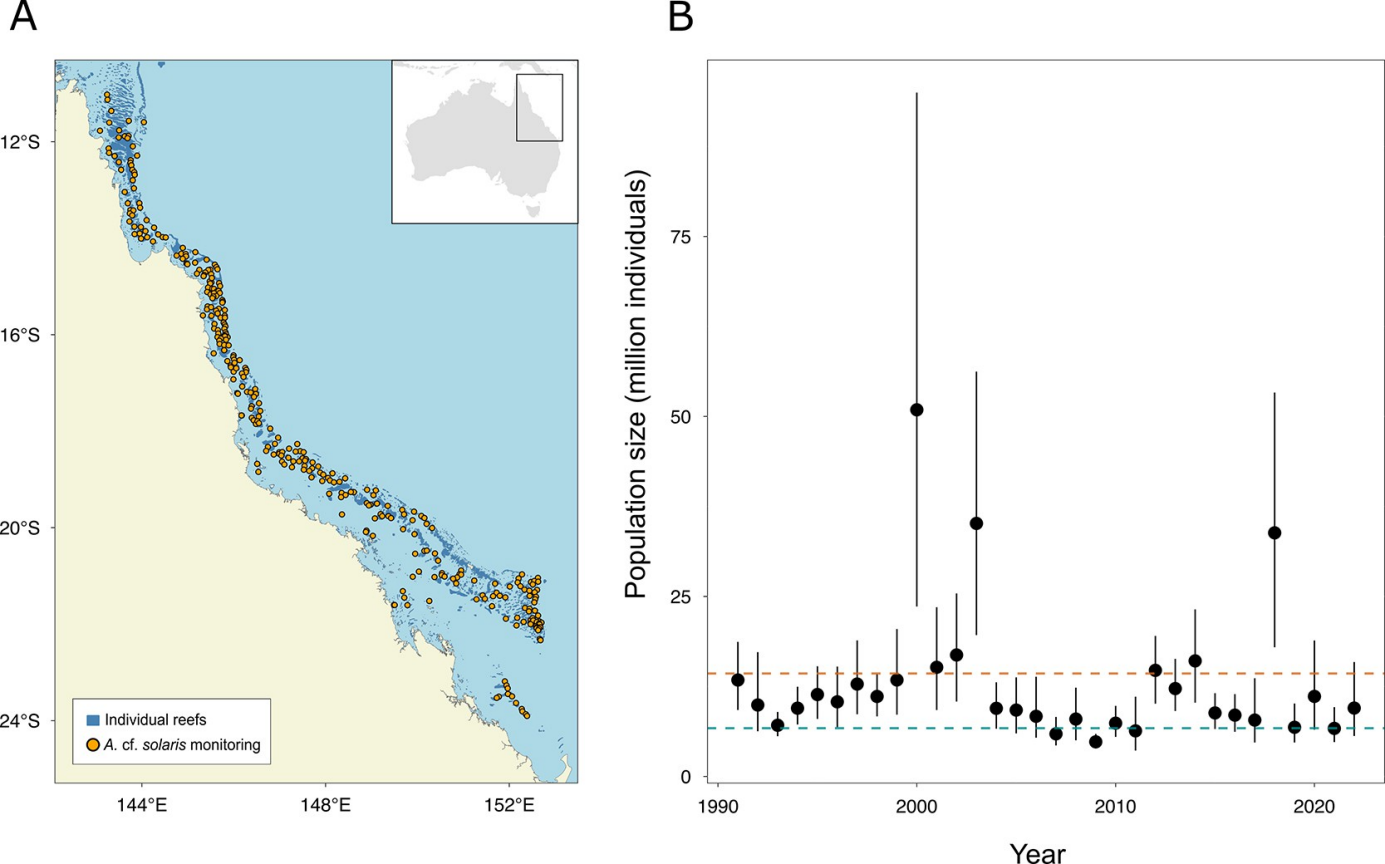
Recent adult *Acanthaster* cf. *solaris* abundance estimates in the Great Barrier Reef (GBR) based on long-term monitoring data. (A) Individual reefs monitored between 1991 and 2022; (B) Contemporary abundance estimates of adult *A*. cf. *solaris* across the GBR (3,054 reefs). For each surveyed reef, individual *A*. cf. *solaris* counts were converted into a density estimate (individuals km^-2^), where mean reef-level density was calculated for each annual sample and a 95% confidence interval of annual mean densities was calculated from 500 pseudo-samples generated by bootstrap for each year. The confidence limits and the mean of the annual mean densities were multiplied by the total surface area of the preferred *A*. cf. *solaris* habitat of reef-building corals on the GBR to calculate census population size. Black dots indicate the average abundance, while vertical lines indicate the extent of the 95% confidence intervals of the 500 mean annual values estimated by bootstrap sampling. The blue and red dashed lines represent, respectively, the harmonic means of the 2.5^th^ (6.7 million) and 97.5^th^ percentiles (14.3 million) of mean annual values over 32 years of monitoring. https://geoportal.gbrmpa.gov.au/.

To better understand the ecological factors constraining *N*_*e*_, we drew upon observational density estimates from 32 years of long-term monitoring surveys to calculate contemporary adult *N*_*c*_ in the GBR with much higher certainty than is typically feasible for marine invertebrate taxa (e.g., [8]). Increased certainty is not only due to the monitoring technique, which allows for rapid surveys of adult populations over large reef areas, but also due to high-resolution spatial mapping enabling reliable estimates of the extent of coral habitat sustaining adult *A*. cf. *solaris* populations. Between 37 and 136 individual reefs were monitored annually between 1991–2022 across the GBR (Fig 4A). Contemporary *N*_*c*_ estimates across 14,199 km^2^ of coral habitat varied between 6.7 and 14.3 million non-cryptic individuals (harmonic means of the annual 2.5^th^ and annual 97.5^th^ percentiles of bootstrap distributions, Fig 4B). Population sizes were 3 to 6 times higher (20–90 million individuals) during outbreaking years (Fig 4B). Considering these *N*_*c*_ confidence intervals, harmonic mean *N*_*e*_*/N*_*c*_ ratios ranged between 0.0047–0.048 applying equilibrium *N*_*e*_ (*N*_*e*_*/N*_*c*_ = 0.022–0.048), historical *N*_*e*_ (*N*_*e*_*/N*_*c*_ = 0.014–0.030) and recent *N*_*e*_ (*N*_*e*_*/N*_*c*_ = 0.0047–0.010).

## Discussion

Lewontin’s paradox arises from comparisons of genetic diversity (π) and census population sizes (*N*_*c*_) across the tree of life. Yet, partitioning the determinants of π and characterising species-specific *N*_*c*_ is a difficult challenge, especially for large and fluctuating populations [4,7,9]. In this study, we estimate the germline mutation rate (*μ)* in *Acanthaster* cf. *solaris* crown-of-thorns sea stars and provide empirical estimates of adult *N*_*c*_ to interrogate the determinants of π in this species. Based on direct observations of 63 *de novo* mutations (DNMs) across 14 pedigree trios, the *A*. cf. *solaris* mean *μ* was 9.13 x 10^−09^ DNMs per-site per-generation (95% CI: 6.51 x 10^−09^ to 1.18 x 10^−08^). Our estimated *μ* exceeds previously reported values for highly abundant terrestrial and freshwater invertebrates (Fig 2). Instead, the *A*. cf. *solaris μ* aligns more strongly with values reported for vertebrate taxa, with largely overlapping 95% confidence intervals with mammals and marine fishes (Fig 2). To provide insight into the magnitude of genetic drift influencing π in large populations experiencing frequent outbreaks, we used our new *μ* estimate to infer historical *N*_*e*_ alongside more recent *N*_*e*_ estimates (that are independent of *μ*). We find that historical (*N*_*e*_ ~ 180,000) and recent *N*_*e*_ (*N*_*e*_ ~ 70,000) estimates are 1–2 orders of magnitude lower than typically reported for highly dispersive marine organisms (e.g., ~ 10^6^, [25,80]) and between 2–3 orders of magnitude lower than *N*_*c*_ estimated from the Great Barrier Reef (GBR) (Fig 4). Our findings of elevated *μ* and reduced *N*_*e*_ in *A*. cf. *solaris* are consistent with *μ* evolving in response to reduced *N*_*e*_ (i.e., drift-barrier hypothesis), suggesting that short periods (~2–3 generations) of outbreaking population sizes exceeding 20–90 million individuals do not allow for lasting effects of selection against high mutation rates. Consistently, *A*. cf. *solaris* exhibits low *N*_*e*_*/N*_*c*_ values (0.0047 to 0.048), indicating significant genetic drift and weak influences of contemporary demographic outbreaks on long-term π. More broadly, our findings suggest that larger contributions of new mutations to π may underlie high mutational loads observed in some marine invertebrate taxa (e.g., bivalves) [39] and may help explain high polymorphism levels in marine populations despite demographic declines [4]. Our study also provides new data valuable for further testing hypotheses about the determinants of mutation rate variation across diverse animal phyla, which are discussed below.

### Low long-term *N*_*e*_ and high *μ* shape genetic diversity in crown-of-thorns sea stars

Using our new *μ* estimate and π from 14 parental genomes (π = 0.0108), the *A*. cf. *solaris* long-term *N*_*e*_ was ~296,000 based on equilibrium expectations (π*/4μ*). Reconstructions of historical *N*_*e*_ in MSMC2 returned a lower harmonic mean *N*_*e*_ ~180,000 over the last 10,000–1 million years and revealed significant impacts of historic climactic changes on *A*. cf. *solaris* long-term *N*_*e*_. We show that *A*. cf. *solaris* have experienced large population fluctuations over the past 1 million years, with the most recent *N*_*e*_ minimum coinciding with the lowest global sea levels during the late Pleistocene (*N*_*e*_ ~ 150,000) [79] (Fig 3). Applying the GONE method, which does not rely on *μ* estimates, recent *N*_*e*_ values were even smaller (< 70,000) considering contemporary populations over last 10–80 generations. While *N*_*e*_ estimates in *A*. cf. *solaris* in the present study (~70,000–180,000) are similar or larger than for most terrestrial chordates (e.g., < 10^5^,) [16], the *N*_*e*_ range reported here is 1–2 orders of magnitude lower than for many highly dispersive marine organisms (~10^6^) [16,25,80], and terrestrial insects (e.g., *Chironomus* midges ~2.16–3.95 x 10^6^, [81]; ~1.4 x 10^6^, *Drosophila*, [77]; ~2 x 10^6^, *Heliconius*; [82]). This finding adds to empirical evidence that extremely abundant marine taxa, including those with outbreaking tendencies, can sustain low *N*_*e*_ that is strongly decoupled from contemporary *N*_*c*_, contradicting the broad notion that large marine populations are immune to the effects of genetic drift [26,83].

From a long-term evolutionary perspective, our findings of reduced *N*_*e*_ and elevated *μ* in *A*. cf. *solaris* provide new genomic results consistent with the drift-barrier hypothesis as an explanation for *μ* variation across taxa. The drift-barrier hypothesis predicts that the efficiency of selection on DNA repair and replication machineries is negatively correlated with the strength of genetic drift. Because the power of genetic drift is inversely proportional to *N*_*e*_ (~1/2*N*_*e*_ for diploid organisms), high mutation rates are expected to evolve in small *N*_*e*_ species, such that π is constrained across diverse taxa (Fig 1) [17,18]. Indeed, negative correlations between per-generation *μ* and *N*_*e*_ are evident across the tree of life [16,17] and recent work demonstrates that environmental constraints on *N*_*e*_ in prokaryotes can rapidly evolve elevated mutation rates, pointing to evolutionarily labile *μ* influencing π [19]. Thus, under the drift-barrier hypothesis, low *N*_*e*_ in *A*. cf. *solaris* (i.e., similar to terrestrial vertebrates) allows for a high *μ* to evolve because selection’s ability to maintain high-fidelity replication and repair mechanisms is compromised (relative to the magnitude of genetic drift as reflected by *N*_*e*_). This observation supports a dominant role of genetic drift in evolutionary change and helps to explain Lewontin’s paradox by describing the stability of π across diverse phyla as a result of the inverse relationship between *μ* and *N*_*e*_, rather than a reflection of relatively constant *N*_*e*_ [17]. With our study, it becomes clear that knowledge of temporal population size fluctuations should be incorporated into investigations of *μ* variation and Lewontin’s paradox to support interpretations of the processes controlling genetic diversity in natural populations [9].

The low *N*_*e*_ values observed in this study likely reflect historical bottlenecks or *N*_*c*_ minima during non-outbreaking periods. While periods of massive *N*_*c*_ experienced by *A*. cf. *solaris* increase opportunities for genetic diversity to be reshuffled through phases of high recombination, rare variants are ultimately lost during repeated population bottlenecks and genetic diversity can only be recovered through the generational input of new mutations or migration [10,30]. Thus, if population size changes occur at a rate higher than coalescent events (that reflect genetic drift), long-term *N*_*e*_ becomes independent of short-term demographic dynamics and contemporary drift [84,85]. As such, frequently fluctuating *A* cf. *solaris* populations are expected to remain far from mutation-drift equilibrium for longer periods of time [86] and a weak relationship is predicted between π and *N*_*c*_ [4]. Consistent with this hypothesis, our calculations of recent adult *N*_*c*_ resulted in low harmonic mean *N*_*e*_*/N*_*c*_ values (0.0047 to 0.048), indicating stronger-than-expected genetic drift and weak influences of extreme population outbreaks on long-term π. It is important to note that *N*_*c*_ upper bounds reported here were below the distribution of *N*_*c*_ estimates for other echinoderms (~10^8^−10^12^; [8]). Because we focused on coral habitats as defined by remote-sensing within the GBR [75], considering the full-distribution of *A*. cf. *solaris* throughout its range in the Pacific Ocean would have likely resulted in species-wide abundance estimates up to 5 x greater than our reported mean *N*_*c*_. We obtained *N*_*e*_ estimates from a sampled population representing a small proportion of the *A*. cf. *solaris* range. However, based on absent spatial population structure in microsatellite data [87], we can infer that gene flow is high across the GBR and that *N*_*e*_ estimates in our present study are likely representative of the population within this region. Thus, while our *N*_*e*_*/N*_*c*_ estimates are conservative for the species-range, we consider them to be precise for the panmictic *A*. cf. *solaris* population within the GBR [87] for which we present genomic data.

In the context of low *N*_*e*_, larger contributions of new mutations to π may thus, in part, explain how moderate polymorphism levels (π = 0.0108) are maintained in *A*. cf. *solaris*, and may explain extreme polymorphism levels in marine bentho-pelagic taxa more broadly (e.g., tunicates and mussels) [4,33,68]. The combination of low *N*_*e*_ and elevated *μ* in *A*. cf. *solaris* (relative to terrestrial invertebrates; Fig 2) could also reconcile evidence for higher observed mutational loads in marine invertebrates compared to terrestrial taxa and marine fishes [39]. Populations with small *N*_*e*_ are expected to hold higher burdens of weakly deleterious mutations due to less efficient purifying selection [88]. For example, high mutation rates in marine invertebrates have been speculated from evidence for elevated genetic loads in Pacific oysters [38]. Similarly, low-frequency deleterious mutations and extreme *dN/dS* ratios in the flat oyster, *Ostrea edulis*, are posited as outcomes of large mutational inputs coupled with small *N*_*e*_ [37]. While we provide evidence for a high germline mutation rate in *A*. cf. *solaris*, the 95% confidence intervals were not distinctly greater than terrestrial animals or marine fishes for which we have *μ* estimates (Fig 2). This result implies that extrinsic processes sustaining reduced *N*_*e*_ (e.g., sweepstakes reproduction or demographic fluctuations) may have a more predominant role, than mutation rate alone, in shaping genetic load and π in *A*. cf. *solaris* and other marine invertebrate populations.

Large variance in reproductive success may be necessary to explain extremely low *N*_*e*_*/N*_*c*_ ratios (i.e., < 1%) in *A*. cf. *solaris* and other wild populations of exploited fish and bivalves [26,34,37,80,89,90]. High skews in reproductive success may be especially relevant for shaping *N*_*e*_ in *A*. cf. *solaris* during non-outbreaking periods when populations can reach densities as low as ~ 3 individuals ha^-1^ [47,48]. Alternatively, asexual reproduction (or budding) of larvae observed in *A*. cf. *solaris* under some circumstances in the laboratory [91], may, even if occasionally, contribute to patterns of genetic diversity observed in natural CoTS populations (reviewed in [92]). Although there is limited support for this hypothesis from genetic simulations [93], an analysis of microsatellites from > 3700 individuals showed no effect of potential clonality on population genetic structure [94]. However, testing these hypotheses, and whether reaching known Allee effect thresholds may amplify reproductive variance [95] or define lower genetic diversity limits required to escape extinction [4] is challenging to evaluate in marine populations [80]. Comparisons of mutation rate and *N*_*e*_ estimates with closely related species that do not experience cyclical outbreaks of a similar nature (e.g., *Acanthaster brevispinus*) or with different mating systems (e.g., brooding Asteroids) would be informative to tease apart the relative contributions of mating strategies (e.g., [16]) versus ecologically-driven *N*_*e*_ fluctuations to *μ* variation.

### Alternative hypotheses for mutation rate evolution in *A*. cf. *solaris*

There is growing evidence that differences in generation time length (e.g., [16]) and reproductive longevity (e.g., [20,21]) are important determinants of germline mutation rate evolution across animal phyla, whereby species with longer generation times have higher *μ* than smaller and shorter-lived organisms [96]. This alternative hypothesis could explain higher *μ* observed in *A*. cf. *solaris* relative to other invertebrate taxa (Fig 2; with the exception of the cyclical parthenogen *Daphnia magna*, 8.9 x 10^−09^, [97]). Compared to annual invertebrates, *A*. cf. *solaris* are longer-lived species (~8 years in the wild; [45]) where most individuals become sexually mature at 2 years of age when individuals become corallivorous [41,98]. In captivity, sexual maturity can be delayed up to 6.5 years if juveniles are confined to herbivory, implying that generation times in natural populations may be longer depending on the availability of live corals as a food resource, although similar delays in sexual maturity have not been confirmed for wild populations. Thus, the mutation rate observed in *A*. cf. *solaris* (values closer to vertebrate taxa; Fig 2) suggests that marine invertebrate taxa may not be an exception to generation time as a predictive factor, despite their phylogenetic distance and unique life history characteristics that differentiate them from most chordates, such as extreme fecundity, large population sizes and bentho-pelagic life cycles [83].

By assigning DNMs to their parental origin, we showed no significant differences in the proportion of male-to-female contributions (α~1) to germline mutations in *A*. cf. *solaris*. This result is consistent with similar male and female mutational contributions observed in ectotherm vertebrates (e.g., reptiles mean α = 1.5; fishes, mean α = 0.8; [16]), and in contrast to stronger male biases observed in mammals that experience larger numbers of mitotic cell divisions during the father’s reproductive lifetime [16,20,58]. This observed variation among ectotherms and mammals may be explained by differences in gametogenesis. Seasonally breeding reptiles and fishes produce gametes during limited periods proceeding mating or spawning activity [99,100], such that differences in the number of male and female cell divisions are expected to be reduced and therefore α is closer to 1 (e.g., [40]). Similar to fishes, in *A*. cf. *solaris* and other echinoderms, highly fecund females produce > 100 million oocytes per season [101] and undergo gametogenesis every spawning period (e.g., November to January; [102]). Although little is known about germline formation in sea stars, the source of germline stem cells (gonia) is likely present throughout the year [103,104], while gametes appear to be reabsorbed after the spawning period [105,106]. The annual renewal of oogonia after the spawning season implies that females replenish germ cells each gametogenesis cycle [104] and that males and females undergo similar numbers of cell divisions throughout their reproductive life spans. In the present study, we show that α estimates for *A*. cf. *solaris* are consistent with theory and empirical data for seasonally reproducing animals. The effect of parental age on the observed mutation rate variation among trios (Fig D in S1 Text) is not clear, however, because unlike other pedigree studies (e.g., [16]), we do not have information about the parental age of wild-caught *A*. cf. *solaris*.

### Deep sequencing supports new inferences for non-model bentho-pelagic marine invertebrates

By sequencing 3-day old larvae, we demonstrate that it should be feasible to detect germline mutations in non-model bentho-pelagic organisms with long-lived planktotrophic larvae if pedigreed larvae can be maintained to the earliest developmental stages preceding planktotrophy (e.g., day 3 larvae)—the key stage impeding successful captive rearing of many bentho-pelagic species. The next main challenge in estimating genomic mutation rates is identifying extremely rare, new mutations against a background of possible sequencing errors [107]. In the present study, we used deep sequencing (60x) of many trios (>10) and applied stringent bioinformatic criteria to detect germline mutations with high certainty. First, we applied best practises workflows with conservative quality thresholds (e.g., genotype quality >70; [59]) that enabled us to reliably compare the *A*. cf. *solaris μ* with most metazoan estimates [16]. Second, we mitigated possible limitations imposed by reference genome quality (e.g., [77]) by visually inspecting BAM files and applying stringent criteria to validate individual DNM candidates. Third, our approach of screening DNMs against all sequenced trios and an independent population genomic *A*. cf. *solaris* dataset helped ensure that DNMs did not correspond to known alleles segregating in contemporary GBR populations. These measures were necessary because larval resequencing was not feasible for independent validation. Additionally, our study examined only single base pair mutations and excluded indel variants, copy number and larger structural variants that may also be important components of germline mutations as the raw material for evolution (e.g., [20,81]). Although our strict approaches for classifying DNMs may have resulted in underestimations of the *A*. cf. *solaris* mutation rate, we have high certainty in the identified DNMs and thus confidence in the resulting patterns, such as the co-occurrence of DNMs between siblings and trends in parental bias. Our conservative approach strengthens our main result that mutational rates in *A*. cf. *solaris* crown-of-thorns sea stars are high relative to other measured invertebrate taxa.

## Supporting information

S1 Text More detailed methods with Figs A-E. **Fig A.** Summary of *A*. cf. *solaris* fertilisation workflow and larval stages. (A) *A*. cf. *solaris* adult being dissected to extract gonads; (B) Female gonads after rinsing in FSW; (C) Mature eggs recently dislodged from the gonad tissue; (D) Fertilised eggs under the microscope presenting a clear fertilisation membrane; (E-I) Larval developmental stages. For each parental cross, we selected two larvae in the early (F) or mid (G) bipinnaria stage (Day 3–8) and early-mid brachiolaria stage (Day 8–9) (H). **Fig B.** Relationship between manta tow counts and SCUBA swim counts from [108]. The observed data (black dots) are expressed as a per-tow basis (200×12 m, with 2 to 15 tows/transects supporting each observation). The regression model is used to generate deterministic predictions of SCUBA swim counts (line) or stochastic predictions function of the number of tows conducted in a given area (e.g., the perimeter of a reef), illustrated here with 5 (red dots) and 25 tows (green dots). Increasing the number of tows decreases the dispersion around the deterministic model. **Fig C.** Variation in parental origins and mutational types for *de novo* mutations (DNM). (A) Per-trio proportions of DNMs phased to their parental origins and those with unknown phasing. There was no significant difference in the proportion of maternally and paternally inherited phased DNMs among trios (Welch two sample t-test; p = 0.50); (B) Distribution of DNMs classified by mutation type, where mutations resulting in a change from C to any base were classified as CpG sites. **Fig D.** Mutation rate estimates for 14 parent-offspring trios grouped by family. There was no effect of family grouping on between group variance (ANOVA; p = 0.33). Mutation rate data points for Trio 7 siblings are overlayed. **Fig E.** Annotated variants (synonymous, nonsynonymous) and their predicted genomic locations according to the *A*. cf. *solaris* reference genome annotations [55]. There was no significant enrichment of annotation categories based on genome-wide expectations (p>0.05) after corrections for multiple tests.
(PDF)

S1 Table Summary of average genome-wide coverage for all sequenced individuals. (XLSX)

S2 Table Relatedness among all individuals confirming expected parent-offspring relationships. (XLSX)

S3 Table Summary of DNM candidates identified in GATK based on Mendelian violations. (XLSX)

S4 Table Summary of DNM candidates identified in GATK based on Mendelian violations and after filtering for sites with no reads supporting parental alternative alleles (AD = 0). (XLSX)

S5 Table Summary of the number of DNMs candidates passing filtering and validation criteria. (XLSX)

S6 Table Summary of manual validation results for 141 DNMs classified as true positives based on alternative caller validation. (XLSX)

S7 Table Summary of 63 DNM candidates passing manual validation criteria, including parent-of-origin (*pooha*) mutational type and annotation information. (XLSX)

S8 Table Summary of the numbers of DNMs phased to maternal and paternal origins using read-back phasing. (XLSX)

S9 Table Mutation rate estimates and article citations used to generate Fig 2 plot. 95% confidence intervals are shown as reported by authors or omitted if not reported. Highlighted mutation rate estimates are removed from Fig 2.
(XLSX)
