# Supplementary material for: High germline mutation rates, but not extreme population outbreaks, influence genetic diversity in a keystone coral predator
Source: PLoS Genet. 2024 Feb 12;20(2):e1011129. doi: 10.1371/journal.pgen.1011129 (PMC10861045; doi:10.1371/journal.pgen.1011129)
Supplement: S1 Text — Fig A. Summary of A. cf. solaris fertilisation workflow and larval stages. (A) A. cf. solaris adult being dissected to extract gonads; (B) Female gonads after rinsing in FSW; (C) Mature eggs recently dislodged from the gonad tissue; (D) Fertilised eggs under the microscope presenting a clear fertilisation membrane; (E-I) Larval developmental stages. For each parental cross, we selected two larvae in the early (F) or mid (G) bipinnaria stage (Day 3–8) and early-mid brachiolaria stage (Day 8–9) (H). Fig B. Relationship between manta tow counts and SCUBA swim counts from [108]. The observed data (black dots) are expressed as a per-tow basis (200×12 m, with 2 to 15 tows/transects supporting each observation). The regression model is used to generate deterministic predictions of SCUBA swim counts (line) or stochastic predictions function of the number of tows conducted in a given area (e.g., the perimeter of a reef), illustrated here with 5 (red dots) and 25 tows (green dots). Increasing the number of tows decreases the dispersion around the deterministic model. Fig C. Variation in parental origins and mutational types for de novo mutations (DNM). (A) Per-trio proportions of DNMs phased to their parental origins and those with unknown phasing. There was no significant difference in the proportion of maternally and paternally inherited phased DNMs among trios (Welch two sample t-test; p = 0.50); (B) Distribution of DNMs classified by mutation type, where mutations resulting in a change from C to any base were classified as CpG sites. Fig D. Mutation rate estimates for 14 parent-offspring trios grouped by family. There was no effect of family grouping on between group variance (ANOVA; p = 0.33). Mutation rate data points for Trio 7 siblings are overlayed. Fig E. Annotated variants (synonymous, nonsynonymous) and their predicted genomic locations according to the A. cf. solaris reference genome annotations [55]. There was no significant enrichment of annotation categories b [file pgen.1011129.s001.pdf]

S1_Text: Supplementary Materials

Parental Gonad Collection and Fertilisation

Adult *A. cf. solaris* were collected from John Brewer Reef (18°38'S, 147°3'E) in the Great Barrier Reef (GBR; November 2020) and transported to the National Sea Simulator (SeaSim) at the Australian Institute of Marine Sciences (AIMS; Townsville, Queensland). Adult *A. cf. solaris* were kept in temperature controlled (26.5 °C) holding tanks supplied with flow-through unfiltered seawater. The experimental room was temperature controlled and set at 28°C ± 0.5°C and contained larval-rearing tanks supplied with filtered seawater (FSW).

Collection of *A.* cf. *solaris* gonad tissue followed a strict protocol to prevent cross contamination between individuals. After carefully placing the animal on a bench, a small ~1 cm incision was made at the proximal end of each *A.* cf. *solaris* arm using a sterile scalpel blade and a small amount of gonad was extracted using clean forceps and put on a Petri dish (Fig AA). The sex of the individual was visually validated, whereby sperm has a milky appearance and eggs can be seen as individual spheres when the gonad is pressed against the clear surface. While male sea stars were returned to the holding tank, three to four gonadal lobes were removed from each female sea star using forceps and placed in a beaker with 200 mL FSW (Fig AB). Each beaker was covered with aluminium foil to prevent contamination with gonads from other individuals. An additional small fragment of gonad tissue was placed in a labelled 2 mL tube with 100% ethanol for genomic analyses.

The extracted female gonads were taken to the experimental room as soon as possible to keep eggs at 28˚C. Male gonads were collected at a later stage (approximately 20 minutes before egg maturation was completed) by making a 1 cm incision as detailed above. The male gonads were placed into a 6-well plate and covered with a lid until the samples were ready to use. In the aquarium room, ovary lobes were washed with FSW over a 500 µm mesh to remove loose eggs and this procedure was repeated several times until no loose eggs were observed (Fig AB). To induce maturation and the release of eggs for each female gonad, the beaker was filled with 200 mL of FSW and a 1 mL vial of 10^{-4} M 1-Methyl adenine was added and gently mixed with the water. This mixture was left to rest for about 40-70 minutes. During this time, mature eggs dislodged from the gonad and sank to the bottom (Fig AC). Once the eggs matured, they were rinsed through a 500 µm mesh to

remove unshed eggs and connective tissues and placed in separate beakers for each individual female with about 500 mL FSW.

A sperm solution was made by gently pressing male gonads to release the sperm. For each male, 2 μL of sperm were pipetted in separate scintillation vials and mixed with ~20 mL FSW. Two millilitres of this sperm solution were added to the egg stock solution and carefully mixed using a plunger to guarantee a homogenous solution. After about 5 to 10 minutes, aliquots were taken from the solution to check fertilisation rates under the microscope (Fig AD). The egg concentration in the stock solutions were also calculated at this point. The egg stock solution was gently mixed with a plunger to guarantee a homogeneous solution and aliquots were checked under the microscope to validate fertilisation (Fig AD).

Larval Rearing: Culture at 24 hours, 72 hours and 8 days

Fertilised egg solutions for each cross were transferred to two 2 L jars for a final concentration of 5 eggs x mL^{-1} or about ~10,000 eggs per glass jar. After 24 hours, the larvae were checked under the microscope to ensure that most larvae had reached the gastrula stage (Fig AE). An initial sample containing hundreds of larvae was taken and placed in a 2 mL tube with 100% ethanol. The remaining larvae were placed in a new set of clean jars by filtering the culture through individually labelled ~50 μm mesh filters that were placed over a bowl with FSW to prevent the larvae from being damaged. Once all the larvae were filtered, a squirt bottle with FSW was used to push the larvae from the filter into the clean jar, the jars were then filled up with FSW with continued aeration. This procedure was repeated every second day to keep the larvae in good condition.

A second larvae culture sample was taken 72 hours after fertilisation, prior to the start of feeding the larvae. By this time most larvae had reached the early bipinnaria stage (Fig AF). On the third day after fertilisation, the early-mid bipinnaria larvae (Fig AF-AG) started receiving food in the form of a mixture of two algae (*Dunaliella sp* CS-353 and *Tisochrysis lutae* CS-177) administered twice daily at a ratio of 3:2 and final concentration of 5000 cells mL^{-1}. A third sample was taken at day 8 or 9, when most larvae had reached the early brachiolaria to mid-brachiolaria stage (Fig AH-AI).

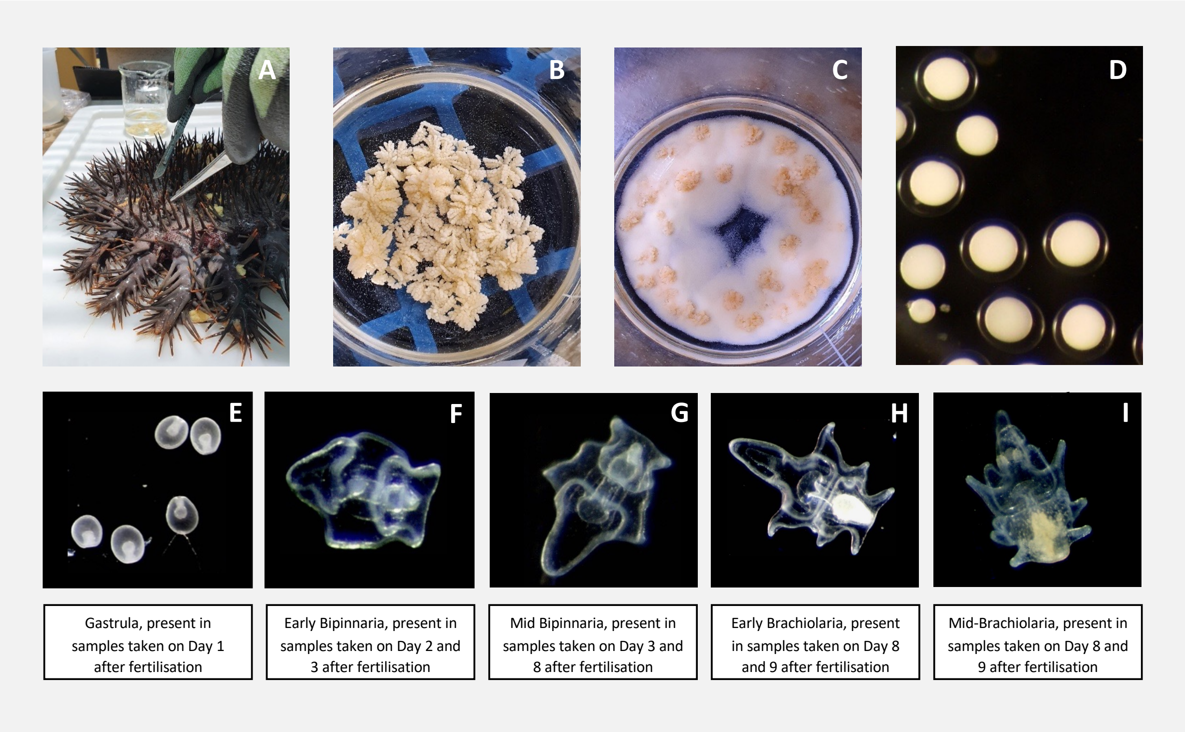

Fig A. Summary of *A.* cf. *solaris* fertilisation workflow and larval stages. (A) *A.* cf. *solaris* adult being dissected to extract gonads; (B) Female gonads after rinsing in FSW; (C) Mature eggs recently dislodged from the gonad tissue; (D) Fertilised eggs under the microscope presenting a clear fertilisation membrane; (E-I) Larval developmental stages. For each parental cross, we selected two larvae in the early (F) or mid (G) bipinnaria stage (Day 3-8) and early-mid brachiolaria stage (Day 8-9) (H). Photo credit: Maria G. Cabrera and Katarina Damjanovic.

DNA extraction, genomic library preparation and sequencing

For each parental cross, we selected two larvae in the early-mid bipinnaria stage (day 3-8) or early-mid brachiolaria stage (day 8-9) (Fig A). DNA from individual larvae was extracted using the QIAGEN Blood and Tissue kit and following modifications for *A.* cf. *solaris* larvae from (1). Adult DNA was extracted from gonad tissue using a modified CTAB protocol optimised for marine invertebrate tissue (2) and purified with PCR-DX Clean beads. Whole genome libraries were prepared for seven biparental families with two larval offspring per family (to generate 14 mother-father-offspring trios including siblings). We used the NEBnext Ultra II FS DNA library preparation kit following manufacturers specifications, with 10-100ng of input DNA and enzymatic fragmentation for 10

minutes to achieve average insert sizes of 300-400 bp. Final amplification consisted of 4-6 cycles as recommended by the manufacturer. Twenty-eight individually dual-indexed genomic libraries were pooled and sequenced on a single lane of the NovaSeq S4 300 cycle to achieve 60X coverage using 2 x 150 bp paired end reads. Sequencing was performed at the Australian Genome Research Facility.

Genomic data processing and mapping

FASTQC was used to examine read quality and adapter contamination (http://www.bioinformatics.bbsrc.ac.uk/projects/fastqc). Raw reads were filtered using Trimmomatic (v 0.39) (3). Adapter sequences were removed using the Illuminaclip option in 'palindrome mode' and reads were trimmed in 4bp sliding windows with a minimum phred score quality of 20 and a minimum read length of 40 bp. Trimmed reads were mapped to the *A.* cf. *solaris* reference genome (GCF_001949145.1_OKI-Apl_1.0; (4); NB: the genome of *A.* cf. *solaris* was incorrectly referred to as *A. planci*; see (5) and (6) for details about name assignment of *Acanthaster* species) using the Burrow-Wheeler Aligner (BWA; version 0.7.17) (7) and the MEM algorithm removing reads with mapping quality < 10. The resulting alignment SAM files were converted to indexed and sorted BAM files using Samtools v1.10 (8). We added read group information to individual BAM files and marked and removed PCR duplicates using picard (http://broadinstitute.github.io/picard/).

Variant calling and site selection

The bioinformatic pipeline used for calling germline mutations from pedigree samples was initially described in (9) and follows best practices principles outlined in (10). All scripts are available on GitHub (https://github.com/lucieabergeron/germline_mutation_rate). Variant calling was performed in GATK v4.0.7.0 and constrained to 693 scaffolds greater than 10,000 bp. Variants were called for each individual and each scaffold separately using HaplotypeCaller in BP-RESOLUTION mode to retain monomorphic sites and associated quality information. The resulting individual gVCF files were collated into a GenomicsDB for each trio using GenomicsDBImport and joint genotyping was performed for each scaffold using GenotypeGVCF. In parallel, the genotypes for all positions (including monomorphic sites) were retrieved from the GenomicsDB and all scaffolds were gathered using GatherVcfs. We analysed relatedness among all individuals to confirm expected parent-

offspring relationships using VCFTOOLS (--relatedness2) (11). We then selected SNPs and performed site-filtering to remove SNPs based on the following site-specific parameters: QD < 2.0; FS > 20.0; MQ < 40.0; MQRankSum < -2.0, MQRankSum > 4.0, ReadPosRankSum < -3.0, ReadPosRankSum > 3.0, SOR > 3.0. These parameters consider the quality of a call at a given site (QD), mapping quality (MQ), strand bias (FS and SOR), mapping quality bias between reference and alternative allele (MQRankSum), and position bias within reads (ReadPosRankSum). Variant quality score recalibration (VQSR) was not performed as this step would likely remove rare variants that are putative *de novo* mutations (DNMs) (9, 10).

Detecting and filtering of de novo mutations

To estimate the candidate DNMs for each trio, we selected only positions identified as Mendelian violations using GATK SelectVariants (-mendelian-violation), where one of the alleles observed in an offspring is not present in either parent. The following filtering criteria were applied to keep only sites in which: (i) The parents were called as homozygous for the reference allele and the offspring was heterozygous (parental alternative allelic depth per site, AD=0); (ii) all individuals within a trio have GQ > 70, DP > 0.5 * average depth of the individual, and DP < 2 * average depth of the individual, where the average depth was calculated based on the VCF files including every position in the genome with a python script *coverage_python.py*; and (iii) the offspring has an allelic balance (AB) between 0.3 and 0.7 meaning that the number of reads supporting the alternative allele is between 30% and 70% of the total reads at this position (only applied to variant sites). Finally, additional quality control was performed by recalling regions with candidate DNMs with bcftools (version 1.2) (12). Any candidates not jointly detected by GATK and bcftools were classified as False Positive (FP) DNMs.

Manual curation of de novo mutations

All DNMs and mutation-associated regions were manually inspected using Integrative Genomics Viewer (IGV) (13) and the original BAM files to rule out mis-mapping errors, validate homozygous parental genotypes and confirm shared DNMs between siblings. We assigned DNMs as spurious if (i) either parent had a one or more reads supporting the alternative allele; (ii) the DNM site showed violations in minimum depth or allelic balance thresholds in offspring or parents; (iii) the DNM was

within 10 bp of an indel; (iv) there was sporadic indel variation or zero coverage zones within 100 bp of the DNM indicating unreliable alignments; (v) there were inconsistencies between parental and offspring genotypes at nearby linked SNPs; (vi) one or more reads supporting the alternative allele were present in any offspring or parents in other trios. As an additional measure to rule out false positive mutations, we compared the remaining DNMs to a large whole-genome dataset of 165 unrelated *A.* cf. *solaris* individuals sampled in the GBR to confirm that sites harbouring DNMs were monomorphic in large populations (Popovic I, unpublished data). For this comparison, we retained singletons and allowed for 50% missing data in the population genomic dataset to capture all reliable polymorphisms.

Germline mutation rate estimation

To estimate the germline mutation rate, we estimated the portion of the genome for which we had power to detect candidate DNMs, considering all sites where mutations could be detected (i.e., number of callable sites) and corrections for the false negative rate. This was done by selecting every position in the VCF files (BP_RESOLUTION output) for which both parents were homozygous for the reference allele and all three individuals passed GQ and DP filters (as described above) (9). A false negative rate was estimated as the proportion of true DNMs that could have been filtered out by sites filters and allelic balance filters applicable to polymorphic positions (9, 14). This FNR would be calculated as:

FNR = 1 - ((1-FNR_RP)*(1-FNR_MQRS)*(1-FNR_FS)*(1-FNR_AB)),

where the first 3 parameters are the expected proportion of sites filtered out by the ReadPosRankSum (FNR_RP), MQRankSum (FNR_MQRS), and FS (FNR_FS) site filters, according to a known null distribution. The FNR_AB is an estimation of the proportion of sites that would be filtered out by the allelic balance filter, estimated as: FNR_AB = number of true heterozygous sites outside the allelic balance threshold / number of true heterozygous sites in the offspring. Here, true heterozygous sites are defined as having one parent homozygous for the reference allele (HomRef), the other parent homozygous for the alternative allele (HomAlt), and the offspring are heterozygous.

The mutation rate was then estimated for each trio as:

μ = (nb_DNM – nb_FP)/(2*C*(1-FNR)),

where nb_DNM is the number of *de novo* mutations, nb_FP is the number of false positive mutations, C is the number of callable sites in the genome and FNR is the false negative rate.

Parental origins and mutation characteristics

De novo mutations were phased to their parental origins using a read-back phasing approach of (15) (https://github.com/besenbacher/POOHA) to determine the proportion of male-to-female contributions (α) to DNMs. DNMs were classified by mutation type and mutations resulting in a change from C to any base were classified as CpG sites. We annotated variants (synonymous, nonsynonymous) and predicted their genomic location (exonic, intronic, intergenic, 5'UTR or 3' UTR, upstream, downstream) with snpEff v5.1 (16) according to the *A.* cf. *solaris* reference genome annotations (4). We assessed whether the number of DNMs in each annotation category was significantly greater than expected by chance. We determined the expected genomic distribution of annotations based on the total number of polymorphic sites per trio, and quantified the probability of observing DNMs for each annotation category using a hypergeometric distribution function *phyper()* in R (17).

Effective population size (N_e) estimation

We used the new germline mutation rate and mean nucleotide diversity across 14 parental genomes as input into the Watterson estimator $\theta = 4N_e\mu$ (18) to estimate long-term N_e. We used ANGSD v0.934 (19) to calculate mean nucleotide diversity. We estimated folded site frequency spectra using *realSFS* and the *saf2theta* option applying a minimum criteria of mapping quality 30, base quality 30, coverage ≥ 10 reads in 100% of individuals (no missing data). We used the thetastat do_stat option to calculate statistics for each site and in overlapping 50kb windows (10kb step size) across the genome. We obtained genome-wide θ estimates by dividing the raw estimates of pairwise theta with the number of sites (nSites) provided by ANGSD. We calculated θ with population nucleotide diversity (π) and calculated effective population size as $N_e = \pi/(4\mu)$.

We inferred historical changes in N_e (> 10,000 years ago) of phased parental genomes using Multiple Sequential Markovian coalescent (MSMC2) analysis (20, 21) and adapted scripts from Github (https://github.com/iracooke/atenuis_wgs_pub). We created a mappability mask for the reference genome using SNPable (http://lh3lh3.users.sourceforge.net/snpable.shtml) to mask genomic regions where sequence reads could not be uniquely mapped. The genome-wide mask file was converted to a bed file using the makeMappabilityMask.py script provided within msmc-tools package (https://github.com/stschiff/msmc-tools). Variant calling was performed using bcftools *mpileup* and bcftools *call* removing sites with mapping quality <30, base quality <30 and indel variants. The bamCaller.py script from the msmc-tools package (https://github.com/stschiff/msmc-tools) was used to produce per-scaffold VCF and mask files for each individual to avoid regions where read coverage is excessively low (<0.5x genome-wide average) or high (>2x genome-wide average). We also retained only scaffolds greater than 1M bp (n=124) representing ~63% of the genome to reduce computational time. Parental genomes were phased using larval offspring and MSMC input files for each trio were generated using the generate_multihetsep.py script from msmc-tools. MSMC analyses were executed for each pair of phased parental genomes (4 haplotypes) using a single randomly chosen offspring for phasing. We applied the default -p parameter as follows (-p 1*2+25*1+1*2+1*3). A distribution of N_e was obtained for each parental pair applying the mean mutation rate as estimated in this study and a generation time of 2 years (22, 23).

To infer N_e on more recent timescales (< 200 generations ago) and to generate N_e estimates that are independent of our inferred μ, we use the Genetic Optimisation for N_e estimation (GONE) method (24). For this analysis, we used the 14 parental genomes and removed scaffolds less than 1M bp and variants with minimum quality < 30, genotype quality < 30, mean depth below 10 and above 50. We additionally excluded singletons and removed variants with > 10% missing data, resulting in 4,591,501 SNPs. Files were converted to plink MAP and PED formats to generate input files for GONE. We used the default recombination rate of 1 centimorgans per megabase and set the maximum recombination rate between pairs of analysed loci to 0.01 (hc=0.01) as recommended by the authors (24). Each analysis was performed with 50,000 replicates, and default settings for all other parameters. Because the GONE method considers the compounded effects of genetic drift from all previous generations, we calculated the arithmetic mean N_e between the last 10-80

generations to exclude the most recent and distant generations where estimation may not be reliable (24).

Crown-of-thorns sea star long-term monitoring data

Field observations of *A.* cf. *solaris* were obtained from the AIMS Long-Term Monitoring Program (LTMP) (25). The LTMP conducts routine benthic surveys across the Great Barrier Reef Marine Park using the manta tow technique, a standardised procedure allowing for the rapid survey of *A.* cf. *solaris* and corals over large areas of coral reef. The technique consists of towing a snorkel diver at constant speed behind a small boat (26, 27). The observer records from the surface the number of non-cryptic sea stars detected over a period of 2 minutes within a band of approximately 10–12 m along the tow path (depending on boat speed, visibility, reef gradient, distance from the bottom). Individual 2-min tows are maintained parallel to the reef crest and repeated until the entire perimeter of a reef is covered. As such, the manta tow is a cost-effective technique that provides useful information on the broad-scale distribution and temporal dynamics of adult *A.* cf. *solaris* across the GBR (28).

Manta tow survey data were available from January 1991 to October 2022. Over this period (32 years), between 37 and 136 individual reefs were surveyed each year across the entire GBR (331 reefs in total). The monitored reefs varied considerably in size, which is reflected by a highly variable number of tows per reef (from 3 to 137, median=42). Tow-level counts were aggregated at the reef level by dividing the total number of *A.* cf. *solaris* recorded for a reef by the number of tows conducted around its perimeter (i.e., mean number of CoTS per tow).

Calibrated estimates of crown-of-thorns sea star density

While manta tow surveys are cost-effective, they are also prone to bias due to the low sightability of small individuals and those hidden within the reef matrix (29). Hence, comparisons with SCUBA swim transects, where the observer carefully inspects the reef matrix in the search of cryptic individuals, have shown that manta tows consistently undercount available sea stars (28, 29). Comparing manta tow counts and SCUBA swim counts performed over the same area, Moran and De'ath (28) obtained a strong relationship between the two abundance estimates (Fig B) and argued

that, once calibrated, manta tow counts can provide unbiased estimates of SCUBA swim counts. Using their manta tow and SCUBA swim count data, we refine the calibration model and generalise it to predict the density of *A.* cf. *solaris* from the mean number of CoTS per tow recorded on any reef.

Similar to Moran and De'ath (28), we performed a linear regression of SCUBA swim counts (SSCs, response) on manta tow counts (MTCs, predictor) after a cube-root-transformation of both variables expressed as per-tow basis (200×12 m, the dimension of a SCUBA transect, roughly equivalent to a 2-min tow search path). Hence, a variable number of tows/transects underlie the reported counts and were used as weights as in the original regression, thus leading to the same model (R^2=0.913, Fig B, see also equation in (30):

$$SSC^{1/3} = 0.8071 + 1.2008\ (MTC)^{1/3}$$

The predicted *A.* cf. *solaris* density (D) expressed in starfish.km^{-2} is thus obtained with:

$$D = (10^6 / 2400) \times (0.8071 + 1.2008\ (MTC)^{1/3})^3$$

The uncertainty around density estimates decreases with the number of conducted tows. We can generate a normally distributed noise around D to reflect the variability of reef-level predictions given the number of tows (N) that were required to survey the perimeter of a reef (Fig B). This is achieved using the R function *rnorm*:

```
D* = rnorm(mean=D, sd=sqrt(se.fit^2+ residual.scale^2)/sqrt(N))
```

where D* is a stochastic prediction of reef-level *A.* cf. *solaris* density, random se.fit is the standard error of the mean prediction (D) and residual.scale the standard deviation of the residuals. It is important to note that SCUBA swim searches are not 100% bias-free (underestimating 'true' density by ~11%, (29)) so that these predictions cannot be considered as absolute estimates of *A.* cf. *solaris* abundance. However, one can reasonably assume they provide accurate estimates of the density of diurnal (>15 cm), non-cryptic adult sea stars (28).

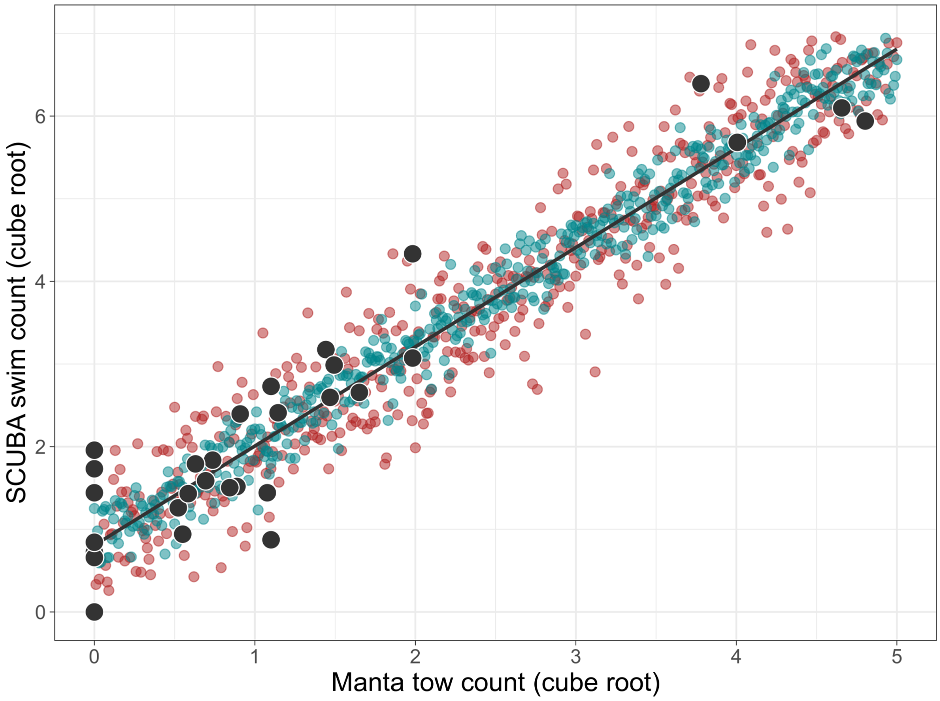

Fig B. Relationship between manta tow counts and SCUBA swim counts from (28). The observed data (black dots) are expressed as a per-tow basis (200×12 m, with 2 to 15 tows/transects supporting each observation). The regression model is used to generate deterministic predictions of SCUBA swim counts (line) or stochastic predictions function of the number of tows conducted in a given area (e.g., the perimeter of a reef), illustrated here with 5 (red dots) and 25 tows (green dots). Increasing the number of tows decreases the dispersion around the deterministic model.

Estimates of reef area and A. cf. solaris habitat

The area of *A.* cf. *solaris* habitat was estimated based on recent high-resolution (10-m) mapping of the GBR geomorphology and substrate type (31). This mapping product characterises the geomorphic zonation of reefs to a depth of 20 m using a specific classification of physical attributes derived from remote-sensing data (sub-surface reflectance, bathymetry, slope angle) and wave modelling. Reef geomorphic zonation is classified into 10 nominal categories defined by expert knowledge and validated with in situ observations. The extent of each geomorphic category is given as 3D surface area calculated from the bathymetric profile, thus providing a more accurate estimate of the actual area of reef habitats. The mapping was initially available for 2,164 offshore reefs of the GBR Marine Park (31) and was latter extended to include 890 fringing and nearshore reefs (32).

As representative *A.* cf. *solaris* habitats, we only considered geomorphic categories that are predominantly covered by consolidated hard substrate, which is more suitable for coral colonisation. According to Roelfsema et al. (31), there are 4 geomorphic categories that are representative of significant 'coral habitat': 'outer reef flat', 'reef slope', 'reef crest', and 'shelter reef slope'. These habitats are likely to support the greatest share of adult *A.* cf. *solaris* populations, as they provide optimal conditions for abundant shelter and food source. The cumulative 3D area of these 4 geomorphic categories across the 3,054 individual reefs amounts to 14,199 km^2 (47.6% of all geomorphic 3D areas). We note that *A.* cf. *solaris* can also be found in habitats deeper than 20 m, and the GBR exhibits significant reef areas below this depth (33). However, the extent of suitable habitat for corals appears relatively limited below 20 m (1/3 of the deep-water reef habitat (34)), especially for tabular *Acropora* corals which are the preferred prey of *A.* cf. *solaris*. High sea star densities are typically observed in areas of rich coral cover, which are usually found around 10-15 m. Thus, reefal areas deeper than 20 m may be considered as marginal habitats for *A.* cf. *solaris*, unlikely to support outbreaking densities (34).

It is important to note that the present definitions of *A.* cf. *solaris* habitats include inshore and outer reef environments where outbreaking *A.* cf. *solaris* densities are less common compared to mid-shelf reefs (35). While defining *A.* cf. *solaris* habitats accurately is challenging, our two estimates of suitable area for *A.* cf. *solaris* (coral-suitable vs. all geomorphic habitats across 3,054 reefs) can be considered as reasonable bounds (14,199 – 29,827 km^2) for the potential extent of significant adult *A.* cf. *solaris* colonisation across the GBR.

Bootstrap re-sampling and confidence limits

We define a sample as the collection of all the reef-level MTCs obtained in any given year *y*, which corresponds to the individual reefs N_y sampled by manta tow during that year. Each reef-level MTC is associated to a number of tows (N_{tows}) conducted along the reef's perimeter. For each year of monitoring, 500 replicate samples were generated by randomly drawing N_y reef-level MTCs with replacement from the corresponding sample. Within each bootstrap sample, a stochastic prediction of reef-level density D* was generated from each drawn value of reef-level MTC and associated N_{tows}

using the calibration model. In doing so, we introduced some variability around density predictions that reflects the uncertainty in detecting *A.* cf. *solaris* from manta tows (consistent with the calibration model). Finally, we averaged the N_y density predictions to estimate the mean reef-level density of non-cryptic *A.* cf. *solaris* per bootstrap sample. This resulted in a distribution of 500 estimates of mean reef-level density for every year of monitoring.

In a second step, mean reef-level densities were multiplied by the 3D area of the total *A.* cf. *solaris* habitat across all 3,054 reefs. The 2.5^{th} and 97.5^{th} percentiles of the resulting distributions were calculated to produce 95% confidence intervals of the annual mean population size of noncryptic *A.* cf. *solaris* between 1991 – 2022.

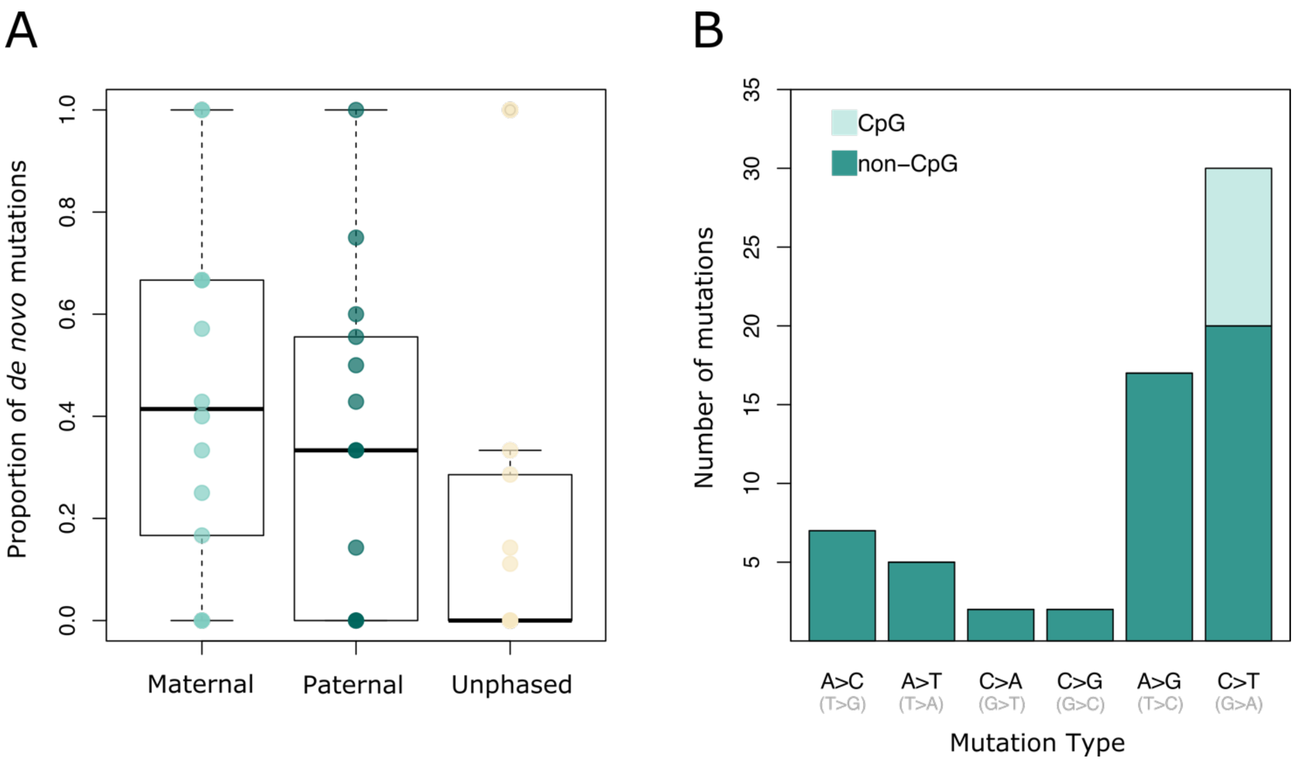

Fig C. Variation in parental origins and mutational types for *de novo* mutations (DNM). (A) Per-trio proportions of DNMs phased to their parental origins and those with unknown phasing. There was no significant difference in the proportion of maternally and paternally inherited phased DNMs among trios (Welch two sample t-test; p=0.50); (B) Distribution of DNMs classified by mutation type, where mutations resulting in a change from C to any base were classified as CpG sites.

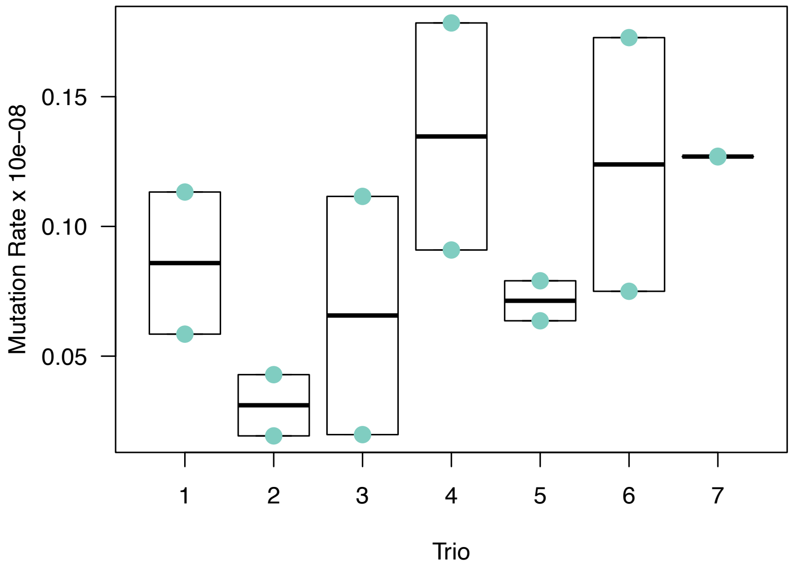

Fig D. Mutation rate estimates for 14 parent-offspring trios grouped by family. There was no effect of family grouping on between group variance (ANOVA; p=0.33). Mutation rate data points for Trio 7 siblings are overlayed.

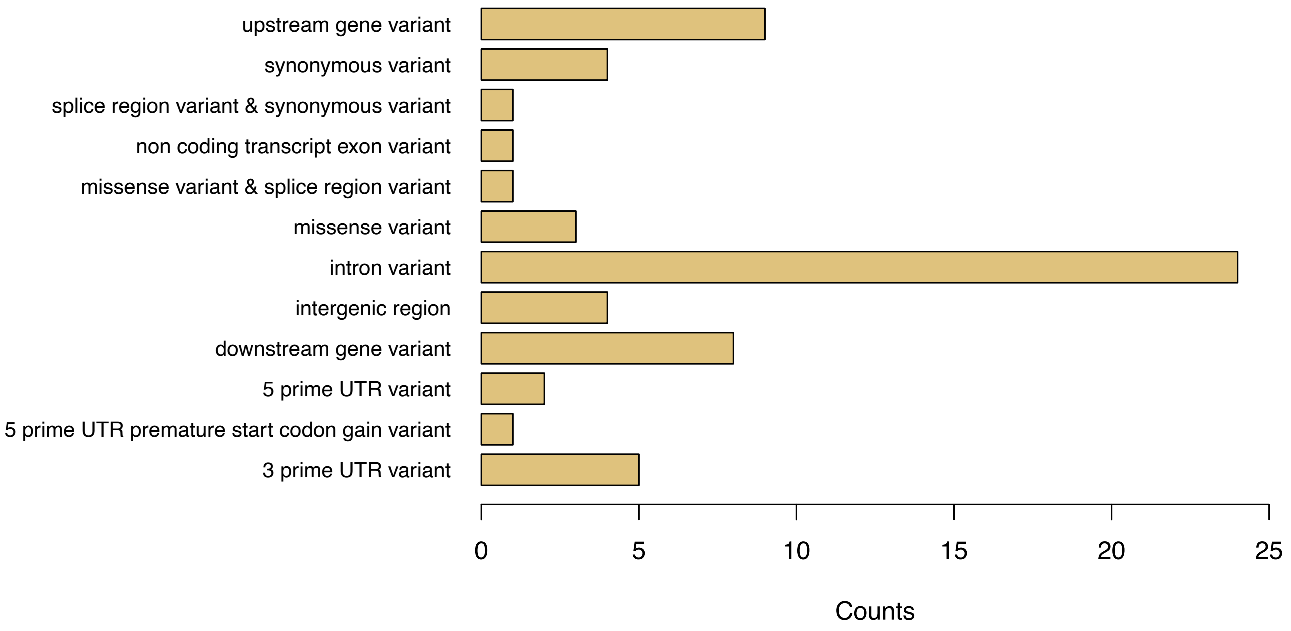

Fig E. Annotated variants (synonymous, nonsynonymous) and their predicted genomic locations according to the *A.* cf. *solaris* reference genome annotations (4). There was no significant enrichment of annotation categories based on genome-wide expectations (p>0.05) after corrections for multiple tests.

References Cited

1. Doyle JR, McKinnon AD, Uthicke S. Quantifying larvae of the coralivorous seastar *Acanthaster* cf. *solaris* on the Great Barrier Reef using qPCR. Marine Biology. 2017;164:1-12.
2. Panova M, Aronsson H, Cameron RA, Dahl P, Godhe A, Lind U, et al. DNA extraction protocols for whole-genome sequencing in marine organisms. Marine genomics: Methods and protocols. 2016:13-44.
3. Bolger AM, Lohse M, Usadel B. Trimmomatic: a flexible trimmer for Illumina sequence data. Bioinformatics. 2014;30(15):2114-20.
4. Hall MR, Kocot KM, Baughman KW, Fernandez-Valverde SL, Gauthier ME, Hatleberg WL, et al. The crown-of-thorns starfish genome as a guide for biocontrol of this coral reef pest. Nature. 2017;544(7649):231-4.
5. Haszprunar G, Spies M. An integrative approach to the taxonomy of the crown-of-thorns starfish species group (Asteroidea: *Acanthaster*): A review of names and comparison to recent molecular data. Zootaxa. 2014;3841(2):271–84-–84.
6. Haszprunar G, Vogler C, Wörheide G. Persistent gaps of knowledge for naming and distinguishing multiple species of crown-of-thorns-seastar in the *Acanthaster planci* species complex. Diversity. 2017;9(2):22.
7. Li H. Aligning sequence reads, clone sequences and assembly contigs with BWA-MEM. arXiv preprint arXiv:13033997. 2013.
8. Li H, Handsaker B, Wysoker A, Fennell T, Ruan J, Homer N, et al. The sequence alignment/map format and SAMtools. bioinformatics. 2009;25(16):2078-9.
9. Bergeron LA, Besenbacher S, Bakker J, Zheng J, Li P, Pacheco G, et al. The germline mutational process in rhesus macaque and its implications for phylogenetic dating. GigaScience. 2021;10(5):giab029.
10. Bergeron LA, Besenbacher S, Turner T, Versoza CJ, Wang RJ, Price AL, et al. The Mutationathon highlights the importance of reaching standardization in estimates of pedigree-based germline mutation rates. Elife. 2022;11:e73577.
11. Manichaikul A, Mychaleckyj JC, Rich SS, Daly K, Sale M, Chen W-M. Robust relationship inference in genome-wide association studies. Bioinformatics. 2010;26(22):2867-73.

12. Li H. A statistical framework for SNP calling, mutation discovery, association mapping and population genetical parameter estimation from sequencing data. Bioinformatics. 2011;27(21):2987-93.

13. Robinson JT, Thorvaldsdóttir H, Winckler W, Guttman M, Lander ES, Getz G, et al. Integrative genomics viewer. Nature biotechnology. 2011;29(1):24-6.

14. Besenbacher S, Liu S, Izarzugaza JM, Grove J, Belling K, Bork-Jensen J, et al. Novel variation and de novo mutation rates in population-wide de novo assembled Danish trios. Nature communications. 2015;6(1):5969.

15. Maretty L, Jensen JM, Petersen B, Sibbesen JA, Liu S, Villesen P, et al. Sequencing and de novo assembly of 150 genomes from Denmark as a population reference. Nature. 2017;548(7665):87-91.

16. Cingolani P, Platts A, Wang LL, Coon M, Nguyen T, Wang L, et al. A program for annotating and predicting the effects of single nucleotide polymorphisms, SnpEff: SNPs in the genome of Drosophila melanogaster strain w1118; iso-2; iso-3. fly. 2012;6(2):80-92.

17. Team RDC. R: A Language and Environment For Statistical Computing. Vienna, Austria: R Foundation for Statistical Computing. 2022.

18. Watterson G. On the number of segregating sites in genetical models without recombination. Theoretical population biology. 1975;7(2):256-76.

19. Korneliussen TS, Albrechtsen A, Nielsen R. ANGSD: analysis of next generation sequencing data. BMC bioinformatics. 2014;15(1):1-13.

20. Schiffels S, Durbin R. Inferring human population size and separation history from multiple genome sequences. Nature genetics. 2014;46(8):919-25.

21. Schiffels S, Wang K. MSMC and MSMC2: the multiple sequentially markovian coalescent. Statistical population genomics: Humana; 2020. p. 147-65.

22. Lucas J. Growth, maturation and effects of diet in *Acanthaster planci* (L.) (Asteroidea) and hybrids reared in the laboratory. Journal of Experimental Marine Biology and Ecology. 1984;79(2):129-47.

23. Caballes CF, Pratchett MS. Reproductive biology and early life history of the crown-of-thorns starfish. Echinoderms: Ecology, Habitats and Reproductive Biology; Whitmore, E, Ed. 2014:101-46.

24. Santiago E, Novo I, Pardiñas AF, Saura M, Wang J, Caballero A. Recent demographic history inferred by high-resolution analysis of linkage disequilibrium. Molecular Biology and Evolution. 2020;37(12):3642-53.

25. AIMS. (2015). AIMS Long-term Monitoring Program: Crown-of-thorns starfish and benthos Manta Tow Data (Great Barrier Reef). https://doi.org/10.25845/5c09b0abf315a. accessed 14-Jul-2023.

26. Moran P, Bradbury R, Reichelt R. Distribution of recent outbreaks of the crown-of-thorns starfish (*Acanthaster planci*) along the Great Barrier Reef: 1985–1986. Coral Reefs. 1988;7:125-37.

27. Miller I, Jonker M, Coleman G. Crown-of-thorns starfish and coral surveys using the manta tow and SCUBA search techniques: Australian Institute of Marine Science Townsville, Australia; 2009.

28. Moran P, De'ath G. Suitability of the manta tow technique for estimating relative and absolute abundances of crown-of-thorns starfish (*Acanthaster planci L.*) and corals. Marine and Freshwater Research. 1992;43(2):357-79.

29. Fernandes L, Marsh H, Moran P, Sinclair D. Bias in manta tow surveys of *Acanthaster planci*. Coral Reefs. 1990;9:155-60.

30. Moran P, De'Ath G. Estimates of the abundance of the crown-of-thorns starfish *Acanthaster planci* in outbreaking and non-outbreaking populations on reefs within the Great Barrier Reef. Marine Biology. 1992;113:509-15.

31. Roelfsema CM, Lyons MB, Castro-Sanguino C, Kovacs EM, Callaghan D, Wettle M, et al. How Much Shallow Coral Habitat Is There on the Great Barrier Reef? Remote Sensing. 2021;13(21):4343.

32. Castro-Sanguino C, Bozec YM, Condie SA, Fletcher CS, Hock K, Roelfsema C, et al. Control efforts of crown-of-thorns starfish outbreaks to limit future coral decline across the Great Barrier Reef. Ecosphere. 2023;14(6):e4580.

33. Harris PT, Bridge TC, Beaman RJ, Webster JM, Nichol SL, Brooke BP. Submerged banks in the Great Barrier Reef, Australia, greatly increase available coral reef habitat. ICES Journal of Marine Science. 2013;70(2):284-93.

34. Beaman RJ. Assessment of deep-water habitat for crown-of-thorns starfish (COTS) in the Great Barrier Reef. Report to the National Environmental Sciences Program Reef and Rainforest Research Centre, Cairns. 2019;45.

35. Vanhatalo J, Hosack GR, Sweatman H. Spatiotemporal modelling of crown-of-thorns starfish outbreaks on the Great Barrier Reef to inform control strategies. Journal of Applied Ecology. 2017;54(1):188-97.
